# Timing of symptomatic venous thromboembolism after surgery: meta-analysis

**DOI:** 10.1093/bjs/znad035

**Published:** 2023-03-13

**Authors:** Tino Singh, Lauri I Lavikainen, Alex L E Halme, Riikka Aaltonen, Arnav Agarwal, Marco H Blanker, Kostiantyn Bolsunovskyi, Rufus Cartwright, Herney García-Perdomo, Rachel Gutschon, Yung Lee, Negar Pourjamal, Robin W M Vernooij, Philippe D Violette, Jari Haukka, Gordon H Guyatt, Kari A O Tikkinen

**Affiliations:** Faculty of Medicine, University of Helsinki, Helsinki, Finland; Faculty of Health Sciences, University of Eastern Finland, Kuopio, Finland; Faculty of Medicine, University of Helsinki, Helsinki, Finland; Faculty of Medicine, University of Helsinki, Helsinki, Finland; Department of Obstetrics and Gynaecology, Turku University Hospital and University of Turku, Turku, Finland; Division of General Internal Medicine, Department of Medicine, McMaster University, Hamilton, Ontario, Canada; Department of Health Research Methods, Evidence and Impact, McMaster University, Hamilton, Ontario, Canada; Department of General Practice and Elderly Care Medicine, University Medical Centre Groningen, University of Groningen, Groningen, the Netherlands; Faculty of Medicine, University of Helsinki, Helsinki, Finland; Raseborg Health Centre, City of Raseborg, Raseborg, Finland; Departments of Gynaecology and Gender Affirmation Surgery, Chelsea and Westminster NHS Foundation Trust, London, UK; Department of Epidemiology and Biostatistics, Imperial College London, London, UK; Division of Urology/Uro-oncology, Department of Surgery, School of Medicine, Universidad del Valle, Cali, Colombia; Department of Health Research Methods, Evidence and Impact, McMaster University, Hamilton, Ontario, Canada; Department of Surgery, Woodstock Hospital, Woodstock, Ontario, Canada; Department of Surgery, McMaster University, Hamilton, Ontario, Canada; Faculty of Medicine, University of Helsinki, Helsinki, Finland; Julius Centre for Health Sciences and Primary Care, University Medical Centre Utrecht, Utrecht University, Utrecht, the Netherlands; Department of Nephrology and Hypertension, University Medical Centre Utrecht, Utrecht, the Netherlands; Department of Health Research Methods, Evidence and Impact, McMaster University, Hamilton, Ontario, Canada; Department of Surgery, Woodstock Hospital, Woodstock, Ontario, Canada; Faculty of Medicine, University of Helsinki, Helsinki, Finland; Department of Health Research Methods, Evidence and Impact, McMaster University, Hamilton, Ontario, Canada; Department of Medicine, McMaster University, Hamilton, Ontario, Canada; Faculty of Medicine, University of Helsinki, Helsinki, Finland; Department of Urology, University of Helsinki and Helsinki University Hospital, Helsinki, Finland; Department of Surgery, South Karelian Central Hospital, Lappeenranta, Finland

## Abstract

**Background:**

The timing at which venous thromboembolism (VTE) occurs after major surgery has major implications for the optimal duration of thromboprophylaxis. The aim of this study was to perform a systematic review and meta-analysis of the timing of postoperative VTE up to 4 weeks after surgery.

**Methods:**

A systematic search of MEDLINE, Scopus, and CINAHL databases was performed between 1 January 2009 and 1 April 2022. Prospective studies that recruited patients who underwent a surgical procedure and reported at least 20 symptomatic, postoperative VTE events by time were included. Two reviewers independently selected studies according to the eligibility criteria, extracted data, and evaluated risk of bias. Data were analysed with a Poisson regression model, and the GRADE approach was used to rate the certainty of evidence.

**Results:**

Some 6258 studies were evaluated, of which 22 (11 general, 5 urological, 4 mixed, and 2 orthopaedic postoperative surgical populations; total 1 864 875 patients and 24 927 VTE events) were eligible. Pooled evidence of moderate certainty showed that 47.1 per cent of the VTE events occurred during the first, 26.9 per cent during the second, 15.8 per cent during the third, and 10.1 per cent during the fourth week after surgery. The timing of VTE was consistent between individual studies.

**Conclusion:**

Although nearly half of symptomatic VTE events in first 4 weeks occur during the first postoperative week, a substantial number of events occur several weeks after surgery. These data will inform clinicians and guideline developers about the duration of postoperative thromboprophylaxis.

## Teaser

The timing of venous thromboembolism after surgery was modelled based on a new systematic review and meta-analysis of 22 prospective studies that included thousands of VTE events from various surgical fields. For symptomatic VTEs occurring within 4 weeks after surgery, 47 per cent occurred by the first, 74 per cent by the second, and 90 per cent by the third week after surgery. This model offers evidence of moderate certainty that, although half of symptomatic VTE events occur during the first postoperative week, a substantial number occur up to 3 weeks after surgery.

## Introduction

The annual number of surgical procedures performed worldwide exceeds 300 million^1^. Venous thromboembolism (VTE), including deep vein thrombosis (DVT) and pulmonary embolism (PE), represents a serious, and on occasion fatal, complication of surgery. Pharmacological prophylaxis decreases the risk of VTE in surgical patients but also increases the risk of bleeding. The decision to use pharmacological prophylaxis therefore presents a trade-off between a reduction in VTE and an increase in major bleeding.

Crucial issues when considering decisions regarding VTE prevention include the starting time and duration of pharmacological thromboprophylaxis. Understanding the timing of postoperative events is therefore necessary. Owing to limitations in the available data, prominent guidelines^2–7^ on thromboprophylaxis have been unable to provide consistent and actionable guidance on the timing of initiation and duration of thromboprophylaxis. The absence of clear guidance contributes to substantial practice variation within and between centres and countries^8–10^.

A recent systematic review and meta-analysis^11^ reported a statistically non-significant decrease in the rate of any (mostly asymptomatic) VTE when thromboprophylaxis was initiated before surgery (risk ratio 0.77, 95 per cent c.i. 0.55 to 1.08). However, there were only 10 symptomatic VTEs (6 symptomatic VTEs among 938 patients (0.6 per cent) in the preoperative and 4 symptomatic VTEs among 941 patients (0.4 per cent) in the postoperative initiation group), highlighting the fragility of current estimates. A Cochrane review^12^ compared the impact of extended thromboprophylaxis with low molecular weight heparin for at least 14 days with in-hospital-only prophylaxis for abdominal or pelvic surgery procedures. The seven randomized trials the authors included reported a total of only eight symptomatic VTE events. Meta-analysis suggested better VTE reduction with extended prophylaxis (1.0 per cent in the in-hospital-only group *versus* 0.1 per cent in patients receiving extended thromboprophylaxis; OR 0.30, 95 per cent c.i. 0.08 to 1.11). The aim of the present study was to systematically review the incidence of symptomatic venous thromboembolism by postoperative day after surgery.

## Methods

The review protocol was registered before starting work on the systematic review (PROSPERO CRD42021241159), and followed PRISMA^13,14^ and MOOSE^15^ guidance.

### Data sources and searches

With the aid of an information specialist, comprehensive searches were performed for studies in general/gastrointestinal, urological, and gynaecological (not obstetric) surgery, without language restrictions, in the MEDLINE, Scopus, and CINAHL databases to search for potentially eligible articles published between 1 January 2009 and 1 April 2022 (Appendices*S1*-3). The review team manually searched reference lists of the included articles and systematic reviews.

### Eligibility criteria

Prospective studies were included if they recruited all patients from the year 2000 or later, in which at least 95 per cent of patients underwent a surgical procedure (in general/gastrointestinal, urological and gynaecological (not obstetric), orthopaedic, thoracic, plastic, hand, breast, endocrine and/or transplant surgery) and reported the timing of at least 20 symptomatic, postoperative VTE (or PE or DVT) events within 90 days after surgery.

### Study selection and data extraction

Standardized forms with detailed instructions were developed for screening of abstracts and full texts, risk of bias, assessment of evidence certainty, and data extraction. Pairs of methodologically trained reviewers independently applied the forms to screen study reports for eligibility and extracted data using online-based DistillerSR™ software (Evidence Partners® Inc., Ottawa, Ontario, Canada). The lead author and/or clinician-methodologist adjudicator resolved potential disagreements.

The following data were extracted from all eligible studies: first author; year of publication; country/countries; surgical field/specialties; number of patients; age; sex; proportion of patients with malignant disease; duration of hospital stay; patient recruitment years, and DVT, PE, and VTE events. Data were retrieved from text, tables or figures. When data were only available in figures, they were retrieved by digitalizing from screenshots of figures.

### Analysis

#### Outcomes

The primary outcome was the proportion of cumulative occurrence of VTE up to 28 days (4 weeks) after surgery. Secondary outcomes included: proportion of cumulative occurrence of PE up to 28 days (4 weeks) after surgery; proportion of cumulative occurrence of DVT up to 28 days (4 weeks) after surgery; and proportion of cumulative occurrence of VTE up to 90 days (3 months) after surgery.

If a study reported the timing of DVT or PE events (but not VTE events), DVT or PE events were converted to VTE events using a previously published method^16^. In that study, data were reviewed from 50 studies that reported DVT, PE, and VTE totals. The overlap was estimated from these studies, and then the degree of overlap was applied to estimate the actual numbers of VTEs in studies that provided only separate reports of DVT and/or PE.

### Risk of bias

As methods to evaluate the risk of bias in studies of prognosis are less developed than the methods for RCTs, through discussion and consensus building, and considering previous literature^17–20^, an instrument was developed to categorize individual studies as being at low or high risk of bias (*Table S1*). This instrument includes issues of sampling and representativeness of the population, study type, loss to follow-up, and thromboprophylaxis documentation.

### Estimation of thromboprophylaxis use

The reported incidence of VTE was adjusted for the use of pharmacological and mechanical thromboprophylaxis separately for each study. For patients who received prophylaxis, the reported incidence was multiplied by the relative risk (RR) of thromboprophylaxis for the duration of prophylaxis use^21^. The updated meta-analyses^22^ informed the RR estimates of thromboprophylaxis as follows: for pharmacological prophylaxis (heparin), RR 0.46 for VTE; for any mechanical prophylaxis, RR 0.43 for VTE; and for combination therapy of pharmacological plus mechanical (*versus* pharmacological alone), RR 0.59 for VTE. Finally, it was inferred that preoperative thromboprophylaxis provided no extra benefit for VTE prevention^11^.

For studies that did not report on use of thromboprophylaxis, thromboprophylaxis use was estimated as follows: web-based survey on thromboprophylaxis informed the authors’ decisions (Appendix*S4*); and, if the survey did not include the procedure(s) undertaken in the study, a study that reported thromboprophylaxis for the procedure(s) from the same time interval and procedure was identified (*Tables S2* and *3*).

### Statistical and sensitivity analyses

A Poisson regression model was fitted using number of VTE (and PE/DVT) events as the dependent variable and population size as the offset variable. Splines were used for days (knots on 2, 6, 10, 14, 18, and 22 days) and categorical variables (study) as predictors. The interaction between time and study proved significant (*P* < 0.001) and was included in the model. Cumulative incidence was predicted for each study separately and predictions were pooled using the inverse of variance of predictions as weights. All analyses were carried out using R language and package Epi^23,24^, and figures were plotted with package ggplot2^25^.

Sensitivity analyses were undertaken. First, the pooled analysis included only studies that reported VTE. Second, sensitivity analyses explored what would have happened under various conditions of thromboprophylaxis, in particular assuming: no pharmacological thromboprophylaxis for all patients (with or without 2 days of mechanical prophylaxis); 1 week of pharmacological thromboprophylaxis for all patients (with or without 2 days of mechanical prophylaxis); 2 weeks of pharmacological thromboprophylaxis for all patients (with or without 2 days of mechanical prophylaxis); and 3 weeks of pharmacological thromboprophylaxis for all patients (with or without 2 days of mechanical prophylaxis).

### Quality of evidence

In the GRADE (Grading of Recommendations Assessment, Development and Evaluation) framework for assessing prognosis, a body of observational studies begins as high-certainty evidence (synonymously, evidence certainty or quality of evidence)^26,27^. Several categories of limitations may, however, reduce the certainty of evidence, including risk of bias, imprecision, inconsistency, and indirectness.

## Results

### Literature search and study characteristics

**Fig. 1:**
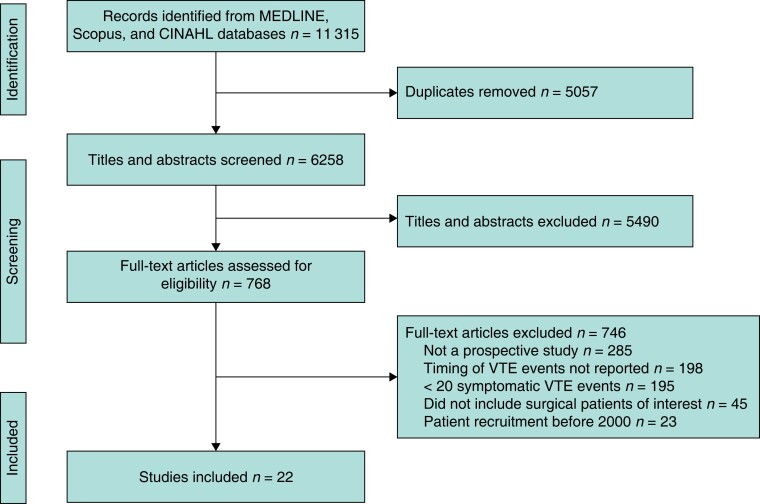
PRISMA flow chart showing selection of articles for review VTE, venous thromboembolism.

Some 6258 titles and abstracts were screened and 768 potentially eligible full-text reports were retrieved, of which 22 studies^28–49^ (with 1 864 875 patients and 24 927 VTE events) proved eligible (*Fig. 1*). Of these 22 studies, 11 included general, 5 urological, 4 mixed, and 2 orthopaedic postoperative populations. The median size of the study population across the studies was 30 468 patients, the median proportion of female patients 49 per cent, the median proportion of patients with malignant disease 85 per cent, and the median duration of hospital stay was 6.5 days (*Table 1*). In 20 of 22 studies^28–30,32–42,44–49^, DVT and PE diagnoses were confirmed by definitive imaging, such as duplex ultrasound examination or CT. In individual studies, the estimated duration of pharmacological thromboprophylaxis varied from 0 to 27 (median 7, i.q.r. 5–11) days, and that of mechanical thromboprophylaxis from 0 to 9 (median 2, i.q.r. 1–2) days (*Table S3*). *Table 1* summarizes the characteristics of the included studies; more details are available in *Tables S2* and *S4*.

**Table 1:** Characteristics of included studies

Reference	Surgical category*	No. of patients	Age (years)†	Female (%)	Malignancy (%)	Duration of hospital stay (days)†	No. of VTE events	Recruitment years
Agnelli *et al.*^43^	Mixed	2373	64	46	100	10	92	n.r.
Kwon *et al.*^31^	General	4195	61	54	39	8	47	2005–2009
Merkow *et al.*^42^	Mixed	44 656	n.r.	65	100	4‡	719	2006–2008
Davenport *et al.*^28^	General	21 943	66	49	100	7‡	446	2005–2009
Shah *et al.*^36^	Mixed	471 867	54	41	20	4‡	7078	2005–2010
Tzeng *et al.*^38^	General	7621	60‡	52	85	6‡	210	2005–2010
Tzeng *et al.*^39^	General	13 771	64‡	52	82	8‡	427	2005–2010
Lavallée *et al.*^32^	Urological	2303	68	21	100	8‡	123	2006–2012
VanDlac *et al.*^40^	Urological	1307	69‡	24	100	8‡	78	2005–2011
Gross *et al.*^41^	General	37 076	66	48	100	10	1018	2005–2010
Moghadamyeghaneh *et al.*^44^	General	116 029	62	52	n.r.	6	4556	2005–2011
Kester *et al.*^45^	Orthopaedic	23 620	NR	61	0	3‡	366	2008–2010
Martin *et al.*^33^	General	3208	64‡	n.r.	100	11‡	161	2005–2012
Moghadamyeghaneh *et al.*^35^	General	219 477	61	52	61	6	2278	2005–2013
Spaniolas *et al.*^37^	General	71 694	45‡	79	0	n.r.	283	2006–2011
Jordan *et al.*^30^	Urological	13 208	61	42	n.r.	4‡	160	2006–2012
McAlpine *et al.*^34^	Urological	65 100	n.r.	n.r.	85	n.r.	956	2006–2014
Benlice *et al.*^48^	General	24 182	43	49	0	8	614	2005–2016
Herforth *et al.*^29^	Mixed	503 602	n.r.	51	n.r.	n.r.	3912	2016
Sager *et al.*^46^	Orthopaedic	39 825	59	42	0	n.r.	102	2005–2017
Merhe *et al.*^49^	Urological	36 753	62	0	100	2‡	423	2008–2015
Kumar *et al.*^47^	General	141 065	57	57	n.r.	1‡	878	2011–2017
Total		1 864 875					24 927	

Details available in supplementary material (*Tables S2* and *S4*). †Mean values are shown, except ‡median. VTE, venous thromboembolism; n.r., not reported.

### Risk of bias and evidence certainty

All studies involved multiple centres and 18 of the 22 studies used consecutive patient recruitment (*Table 2*). In one study it was certain that loss to follow-up was less than 10 per cent. None of the studies accurately reported the proportion of patients receiving thromboprophylaxis, including type and duration of prophylaxis. Overall, 1 study was judged as having a low and 21 studies a high risk of bias (*Table 2*), and so the certainty of evidence was rated down owing to risk of bias. Evidence review raised no concerns regarding imprecision, inconsistency, or indirectness, and therefore a quality rating (evidence certainty) of moderate was warranted.

**Table 2:** Risk of bias

	Sampling and representativeness of population	Documentation of thromboprophylaxis	Follow-up of patients	Study type	Risk of bias
Agnelli *et al.*^43^	+	–	+	+	Low
Kwon *et al.*^31^	+	–	–	+	High
Merkow *et al.*^42^	+	–	–	+	High
Davenport *et al.*^28^	–	–	–	+	High
Shah *et al.*^36^	–	–	–	+	High
Tzeng *et al.*^38^	+	–	–	+	High
Tzeng *et al.*^39^	–	–	–	+	High
Lavallée *et al.*^32^	+	–	–	+	High
VanDlac *et al.*^40^	–	–	–	+	High
Gross *et al.*^41^	+	–	–	+	High
Moghadamyeghaneh *et al.*^44^	+	–	–	+	High
Kester *et al.*^45^	+	–	–	+	High
Martin *et al.*^33^	+	–	–	+	High
Moghadamyeghaneh *et al.*^35^	+	–	–	+	High
Spaniolas *et al.*^37^	+	–	–	+	High
Jordan *et al.*^30^	+	–	–	+	High
McAlpine *et al.*^34^	+	–	–	+	High
Benlice *et al.*^48^	+	–	–	+	High
Herforth *et al.*^29^	+	–	–	+	High
Sager *et al.*^46^	+	–	–	+	High
Merhe *et al.*^49^	+	–	–	+	High
Kumar *et al.*^47^	+	–	–	+	High

+, Low risk; –, high risk.

### Timing of events

**Fig. 2:**
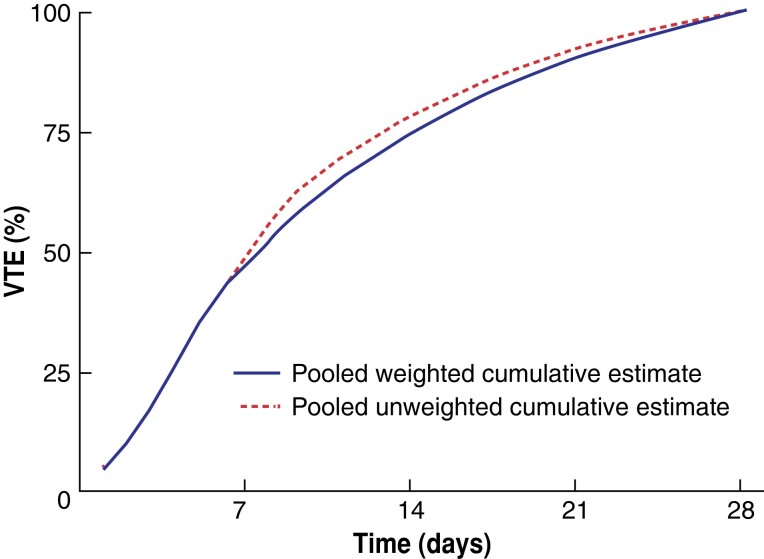
Proportion of cumulative occurrence of venous thromboembolism by time during the first 28 days (4 weeks) after surgery: all included studies pooled VTE, venous thromboembolism.

Regarding the cumulative VTE risk during the first 4 weeks after surgery, 47.1 per cent of the VTE events occurred during the first week, 26.9 per cent during the second week, 15.8 per cent during the third week, and 10.1 per cent during the fourth week after operation (*Fig. 2* and *Table S5*).

**Fig. 3:**
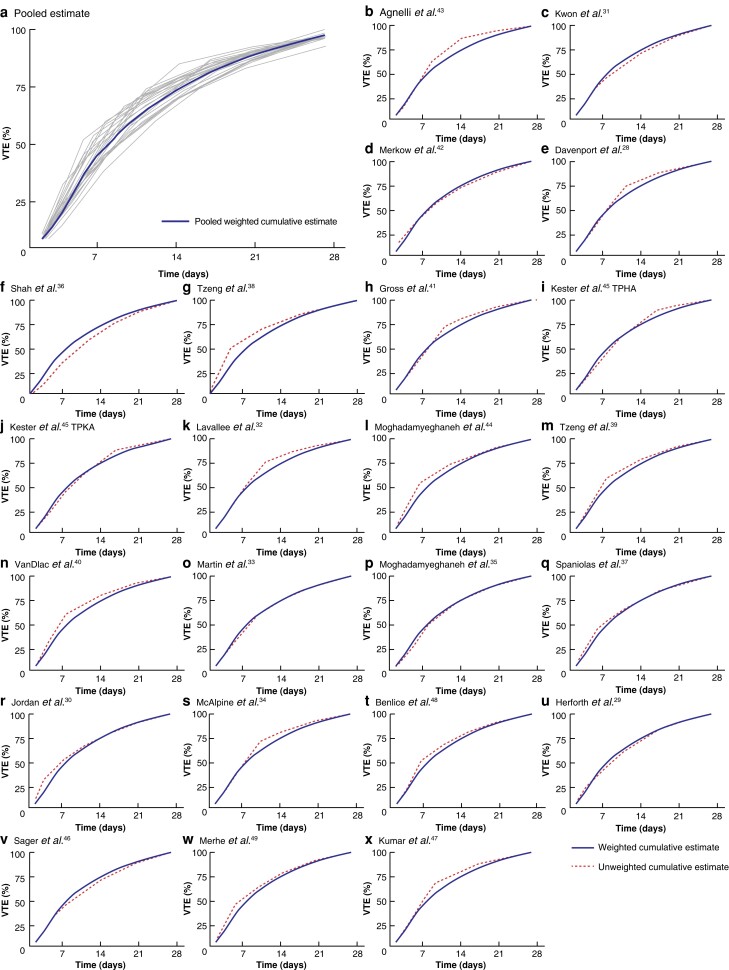
Proportion of cumulative occurrence of venous thromboembolism by time during the first 28 days (4 weeks) after surgery in individual studies. **a** Weighted cumulative estimates (pooled in blue line and individual studies in grey lines) of venous thromboembolism (VTE) occurrence and **b–x** cumulative weighted and unweighted estimates for individual studies. VTE, venous thromboembolism; TPHA, total or partial hip arthroplasty; TPKA, total or partial knee arthroplasty.

The timing of VTE was consistent between individual studies (*Fig. 3*). For instance, from the cumulative VTE risk of 4 weeks, the median estimate of the proportion of VTEs that occurred by 2 weeks was 77.7 (i.q.r. 74.8–80.2) per cent, with highest and lowest estimates of 86.0 and 68.2 per cent (*Fig. 3* and *Fig. S1*).

Data on the timing of PE, DVT, and VTE events separately are provided in *Figs S2–S4*. The sensitivity analyses did not change the results materially (*Figs S5–S7*). No eligible studies reported on VTE up to 90 days, so pooled estimates did not extend beyond 28 days after surgery.

## Discussion

This systematic review and meta-analysis, pooling 22 studies, represents the first available summary of the postoperative timing of symptomatic VTE. The pooled results provide evidence of moderate quality that, of the cumulative VTE risk during the first 28 days (4 weeks) after surgery, 47.1 per cent of the VTE events occur during the first, 26.9 per cent during the second, 15.8 per cent during the third, and 10.1 per cent during the fourth week after operation.

Strengths of this study include a comprehensive search (studies published 2009 or later; patients recruited after 2000). The search was limited to contemporary studies, because the baseline risks of VTE and bleeding have likely changed over time^50–52^. To mitigate the effect of publication bias, studies with at least 20 symptomatic VTE events were included. Teams of two reviewers assessed eligibility and risk of bias, and undertook data extraction with a clinician-methodologist adjudicating discrepancies. A total of 22 prospective studies (each directly providing information regarding timing of VTE in dozens of surgical procedures in various fields of surgery) that included thousands of VTE events (high statistical power leading to high precision for the pooled results) were identified. Considering the use of thromboprophylaxis, the timing of postoperative VTE events was pooled up to 28 days (4 weeks) after surgery, a duration of extended prophylaxis frequently used by clinicians^4,7,53–58^. Studies proved consistent regarding timing of VTE (as well as PE and DVT) and sensitivity analyses yielded results similar to the primary analyses. Applying the GRADE approach to certainty of evidence, the results were judged to provide evidence of moderate certainty.

This study has limitations, reflecting limitations in the available evidence. Because observational studies have less established indexing than randomized trials, some relevant studies may have been missed. Second, most of the included studies used the American College of Surgeons National Surgical Quality Improvement Program (ACS-NSQIP) database, which does not collect data on perioperative thromboprophylaxis. Owing to lack of data regarding thromboprophylaxis, thromboprophylaxis practice was estimated based on the published literature and results of a clinician survey (*Supplementary methods S4*). The finding that sensitivity analyses assuming different thromboprophylaxis regimens did not materially change the results suggests that they are trustworthy. Third, as most studies used the ACS-NSQIP database, some studies included the same procedures, and sometimes even data from the same patient recruitment years, resulting in some double-counting of patients and events. It is unlikely, however, that this double-counting would have seriously biased the results, although it likely led to some degree of false precision. The reason is the striking consistency of results across studies; thus, any double-counting would be of patients with results similar to those of patients counted only once^59^. Finally, owing to the lack of studies reporting this information, it was not possible to pool timing of VTE events beyond the initial postoperative 4 weeks, or report the proportion of patients with more proximal venous thrombosis or embolism.

Little previous work has attempted to summarize the literature informing the timing of postoperative VTE^60–62^. Globally, the first procedure-specific guideline in any field of surgery (urological surgery)^4^ was based on a series of systematic reviews and meta-analyses on procedure-specific risks of VTE and bleeding after urological surgery^16,63^. To be able to account for the timing of VTE^62^, the authors pooled the results from two large studies^60,61^: a prospective study that included British women, of whom almost 900 had VTE within 12 weeks after surgery; and a retrospective study that included US surgical patients with 305 VTE events (172 among patients who had abdominal surgery) within 6 months after operation. VTE events occurred later when the results of these two studies were pooled^60–62^ than in the present work: 27 *versus* 47 per cent respectively of the VTE events occurred by the first week, and 54 *versus* 74 per cent by the second week. One important reason for the earlier occurrence of VTEs in the present study is that the previously published work did not take the use of thromboprophylaxis into account. As thromboprophylaxis is often used only during the first week after surgery^10^, not taking it into account overestimates the proportion of late VTEs as VTEs that would have occurred early and were prevented by prophylaxis are missed, whereas those that occur later when prophylaxis is given less frequently are not missed.

Both the present and previous work^62^ benefited from focusing on symptomatic VTE events. This is especially important because scanning for asymptomatic events (typically at fixed time points such as a week or two post-surgery) would bias the timing (treatment of asymptomatic events would prevent the occurrence of symptomatic events at a later time point) and focus on an outcome that is not important to patients. The present systematic review also benefits from including only prospective studies (not the case for the US study^61^), as retrospective studies often miss VTEs that occur after discharge, and studies with contemporary patient recruitment years, and therefore more up-to-date surgical and perioperative practices (not the case for the British study^60^). These new results therefore represent more accurate and up-to-date estimates of the timing of VTE within the first 28 days after surgery.

The results of this systematic review have important implications for the surgical practice globally. Surgical thromboprophylaxis practice, especially after discharge, varies widely both within and between countries^8–10,64^. The timing and duration of postoperative VTE prophylaxis is a key question in daily clinical practice. Although the evidence establishes that almost half of VTE events in the first month after surgery occur during the first postoperative week, it also demonstrates that a substantial number of VTE events arise during the third, or even fourth, week after surgery. These results suggest the possible importance of extended prophylaxis, especially in patients with high risk of VTE. Although meta-analyses of the randomized trials have failed, owing to insufficient statistical power^11,12^, to establish the optimal starting time and duration of thromboprophylaxis, clinicians and guideline developers can use the results of the present systematic review, together with knowledge of baseline risks of VTE and bleeding, to guide the starting time and duration of thromboprophylaxis.

These results will also prove useful for the planning and conduct of future clinical research, which should benefit from the present identification of limitations in past studies. There were limitations regarding reporting of use, starting time, and duration of thromboprophylaxis, and so data from a contemporary survey of surgeons’ practices on thromboprophylaxis had to be relied on. Because of lack of direct evidence on this issue, most studies were judged as having a high risk of bias, and the certainty of evidence was therefore lowered from high to moderate. Future prospective studies, including use of representative patient populations, clear documentation of VTE, DVT, PE, and their follow-up times, and documentation of thromboprophylaxis used, would improve the evidence base, and consequently further rationalize the global practice of thromboprophylaxis in surgery.

## Supplementary Material

znad035_Supplementary_Data

## Data Availability

The corresponding author is the custodian of the data and will provide access to data on request.
