# Supplementary material for: Timing of symptomatic venous thromboembolism after surgery: meta-analysis
Source: Br J Surg. 2023 Mar 13;110(5):553–61. doi: 10.1093/bjs/znad035 (PMC10364527; doi:10.1093/bjs/znad035)
Supplement: znad035_Supplementary_Data [file znad035_supplementary_data.docx]

**Timing of symptomatic venous thromboembolism after surgery: A systematic review and meta-analysis**

Tino Singh^1,2^, Lauri I. Lavikainen^1^, Alex L.E. Halme^1^, Riikka Aaltonen^3^, Arnav Agarwal^4,5^, Marco H. Blanker^6^, Kostiantyn Bolsunovskyi^1,7^, Rufus Cartwright^8,9^, Herney García-Perdomo^10^, Rachel Gutschon^5,11^, Yung Lee^12^, Negar Pourjamal^1^, Robin W.M. Vernooij^13,14^, Philippe D. Violette^5,11^, Jari Haukka^1^, Gordon H. Guyatt^5,15^ and Kari A. O. Tikkinen^1,16,17^

^1^Faculty of Medicine, University of Helsinki, Helsinki, Finland

^2^Faculty of Health Sciences, University of Eastern Finland, Kuopio, Finland

^3^Department of Obstetrics and Gynecology, Turku University Hospital and University of Turku, Turku, Finland

^4^Division of General Internal Medicine, Department of Medicine, McMaster University, Hamilton, ON, Canada

^5^Department of Health Research Methods, Evidence and Impact, McMaster University, Hamilton, ON, Canada

^6^Department of General Practice and Elderly Care Medicine, University Medical Center Groningen, University of Groningen, Groningen, The Netherlands

^7^Raseborg Health Center, City of Raseborg, Raseborg, Finland

^8^Departments of Gynaecology and Gender Affirmation Surgery, Chelsea & Westminster Hospital NHS Foundation Trust, London, UK

^9^Department of Epidemiology & Biostatistics, Imperial College London, UK

^10^Division of Urology/Uro-oncology, Department of Surgery, School of Medicine, Universidad del Valle, Cali, Colombia

^11^Department of Surgery, Woodstock Hospital, Woodstock, ON, Canada

^12^Department of Surgery, McMaster University, Hamilton, ON, Canada

^13^Julius Center for Health Sciences and Primary Care, University Medical Center Utrecht, Utrecht University, Utrecht, The Netherlands

^14^Department of Nephrology & Hypertension, University Medical Center Utrecht, Utrecht, The Netherlands

^15^Department of Medicine, McMaster University, Hamilton, ON, Canada

^16^Department of Urology, University of Helsinki and Helsinki University Hospital, Helsinki, Finland

^17^Department of Surgery, South Karelian Central Hospital, Lappeenranta, Finland

**Corresponding author.** Kari Tikkinen. Department of Urology, University of Helsinki and Helsinki University Hospital, Biomedicum 2 B, PL 13, Tukholmankatu 8 B, 00290 Helsinki, Finland. E-mail address: [kari.tikkinen@helsinki.fi](mailto:kari.tikkinen@helsinki.fi). **ORCID ID:** 0000-0002-1389-8214. **Twitter:** @KariTikkinen

Supplementary Materials - Index

[Supplementary Methods 4](#_Toc128065487)

[Appendix S1. Supplementary Methods: MEDLINE search history 4](#_Toc128065488)

[Appendix S2. Supplementary Methods: SCOPUS search history 8](#_Toc128065489)

[Appendix S3. Supplementary Methods: CINAHL search history 11](#_Toc128065490)

[Table S1. Design features used for assessment of risk of bias 16](#_Toc128065491)

[Appendix S4. Supplementary Methods: Details regarding thromboprophylaxis estimation 17](#_Toc128065492)

[Table S2. Estimated duration of thromboprophylaxis for each study and surgical procedures. 18](#_Toc128065493)

[Table S3. Estimated thromboprophylaxis duration for each procedure. 20](#_Toc128065494)

[Supplementary Results 24](#_Toc128065495)

[Table S4. Characteristics of the included studies, including information on included surgical procedures. 24](#_Toc128065496)

[Table S5. Proportion of cumulative occurrence of venous thromboembolism by time (days) during the first 28 days (4 weeks) post-surgery (all included studies pooled) 25](#_Toc128065497)

[Figure S1. Proportion of cumulative occurrence of venous thromboembolism by time (days) during the first 28 days (4 weeks) post-surgery in individual studies (landscape version). 26](#_Toc128065498)

[Studies reporting separately pulmonary embolism (PE) and/or deep vein thrombosis (DVT) events 27](#_Toc128065499)

[Figure S2. Proportion of cumulative occurrence of venous thromboembolism by time (days) during the first 28 days (4 weeks) post-surgery, modelled using PE events only. 27](#_Toc128065500)

[Figure S3. Proportion of cumulative occurrence of venous thromboembolism by time (days) during the first 28 days (4 weeks) post-surgery, modelled using DVT events only. 28](#_Toc128065501)

[Figure S4. Proportion of cumulative occurrence of venous thromboembolism by time (days) during the first 28 days (4 weeks) post-surgery (using only studies that reported on VTE events). 29](#_Toc128065502)

[Sensitivity analyses regarding duration of thromboprophylaxis 30](#_Toc128065503)

[Figure S5. Proportion of cumulative occurrence of venous thromboembolism by time (days) since surgery during the first 28 days (4 weeks) post-surgery with 0, 1, 2 and 3 weeks of estimated thromboprophylaxis (including mechanical thromboprohylaxis). 30](#_Toc128065504)

[Figure S6. Proportion of cumulative occurrence of venous thromboembolism by time (days) since surgery during the first 28 days (4 weeks) post-surgery with 0, 1, 2 and 3 weeks of estimated thromboprophylaxis (including mechanical thromboprohylaxis). 31](#_Toc128065505)

[Figure S7. Proportion of cumulative occurrence of venous thromboembolism (main model), pulmonary embolism (PE) and deep vein thrombosis (DVT) by time (days) during the first 28 days (4 weeks) post-surgery. 32](#_Toc128065506)

[References 33](#_Toc128065507)

[Table S3 references 33](#_Toc128065508)

# Supplementary Methods

## Appendix S1. Supplementary Methods: MEDLINE search history

Search Strategy:

--------------------------------------------------------------------------------

1 exp Gynecologic Surgical Procedures/ or exp Genital Diseases, Female/su or exp Genitalia, Female/su or Uterine Artery/su or Gynecology/su or Female Urogenital Diseases/su or Oocyte Retrieval/ or exp Ovarian Cysts/su or exp Pregnancy, Ectopic/su or "Dilatation and Curettage"/ or exp Pregnancy Complications/su or Hysteroscopy/ or exp Pelvic Organ Prolapse/su

2 Urethra/su or Urinary Bladder/su or exp Urinary Incontinence/su or Suburethral Slings/ or Sterilization Reversal/

3 limit 2 to female

4 1 or 3

5 ((gyn?ecolog* or ovar* or vulv* or vagin* or transvagin* or adnex* or fibroid* or myoma or leiomyoma or pelvic or perineal or endometri* or cervic* or cervix or uterus or uterin* or fallop* or curettage or labia* or clitoris) adj3 (resect* or excision* or ablation or conisation or conization or endoscop* or embol* or operation or torsion or amputation or rupture or cytoreduc*or repair or fixation or reconstruct* or surgery or surgeries or procedure* or removal)).mp.

6 (cervicopex* or cervicectom* or colpectom* or colporrhagh or compopex* or colposcop* or colposuspen* or colpotom* or culdoplast* or exenteration* or fibroidectom* or fimbriectom* or hysterectom* or hysteropex* or hysteroscop* or hysterosalpingo* or LAVH or LEEP or LLETZ or USLS or myomectomy* or oophorectom* or omentectom* or ovariectom* or perineoplast* or perineorrhaph* or sacrocolpop* or sacropex* or salpingectom* or salpingostom* or salpingo-oophorectom* or trachelectom* or tubectom* or tuboplast* or uteroscop* or vaginotom* or vaginoplast* or vulvectom* or Wertheim or labiaplast*).mp.

7 (oocyte* adj2 (retriev* or aspirat* or collect*)).mp.

8 (Tubal adj2 (excision or ligation or occlusion or ring or rings or sterilization or reanastomosis)).mp.

9 ((vaginal or pubovaginal or transvaginal or retropubic or transobturator or prolapse) adj3 (tape* or sling* or mesh*)).mp.

10 (TVT or mini-sling or miniarc or bulkamid).mp.

11 or/4-10

12 (Gyneco* adj3 surg*).jw.

13 (excision adj3 loop).mp.

14 ((ovar* or luteum or luteal) adj3 (cystectom* or resect* or enucleat* or excision* or ablation or conisation or conization or endoscop* or embol* or operation or torsion or amputation or rupture or cytoreduc*or repair or fixation or reconstruct* or surgery or surgeries or procedure* or removal)).mp.

15 ((ectopic or extrauterin* or tubal*) adj3 pregnan* adj3 (cystectom* or resect* or enucleat* or excision* or ablation or conisation or conization or endoscop* or embol* or operation or torsion or amputation or rupture or cytoreduc*or repair or fixation or reconstruct* or surgery or surgeries or procedure* or removal)).mp.

16 ((sacrospinous or uterosacral or sacral ligament) adj3 (fixat* or suspens* or plicat*)).mp.

17 ((dilatation or curettage or evacuation) adj5 (conception or cervix or cervic* or uter* or pregnancy or placent*)).mp.

18 or/4-17

19 Appendectomy/ or exp Bariatric Surgery/ or exp Cholecystectomy/ or exp Colectomy/ or exp Gastrectomy/ or Hepatectomy/ or Herniorrhaphy/ or pancreatectomy/ or Pancreaticoduodenectomy/ or pancreaticojejunostomy/ or Splenectomy/

20 General Surgery/ or exp digestive system surgical procedures/

21 exp Digestive System/su or Cholecystitis/su or Gallbladder/su or exp Gallbladder Diseases/su or Hernia, Abdominal/su or Hernia, Inguinal/su or exp Hernia, Ventral/su or exp Intestinal Diseases/su or exp Liver Diseases/su or exp Pancreas/su or exp Pancreatic Diseases/su or Spleen/su or exp Splenic Diseases/su or exp Stomach Diseases/su

22 (appendectom* or appendicectom* or colectomy* or proctocolectom* or cholecystectom* or duodenectom* or gastrectom* or hernioplast* or herniorrhaph* or herniotom* or jejunectom* or pancreatectom* or pancreaticojejunostom* or pancreaticoduodenectom* or duodenopancreatectom*).mp.

23 ((surgery or resection* or excision* or repair* or operation* or laproscop* or laparoscop* or sleeve*) adj3 (abdominoperineal or perineal or anal* or anus or appendix or bowel* or colon* or duoden* or jejun* or ileal* or ileum* or jejuno?ileal or intestine* or gall bladder or gall?bladder or gastric or bariatric* or stomach or hernia or liver or adenoma or hepatoma* or hepatocellular* or rectal* or rectum)).mp.

24 ((general or abdominal or major) adj3 (surgery or surgical)).mp.

25 (prolapse adj3 rectal).mp.

26 (Rectopexy or rectosigmoidectom* or sigmoidectom* or DHoore or d'hoore or Delorme or Altemeier).mp.

27 or/19-26

28 Urologic Surgical Procedures.mp. or exp Urologic Surgical Procedures/

29 Ambulatory Surgical Procedures/

30 Cystectomy.mp.

31 Cystoscopy.mp.

32 Cystostomy.mp.

33 Cystotomy.mp.

34 Kidney Transplantation.mp.

35 Nephrectomy.mp.

36 Ureteroscopy.mp.

37 Urinary Diversion.mp.

38 exp Nephrostomy, Percutaneous/

39 Nephrostomy Percutaneous.mp.

40 exp Circumcision, Male/

41 Circumcision Male.mp.

42 Orchiectomy.mp.

43 Orchiopexy.mp.

44 Penile Implantation.mp.

45 Prostatectomy.mp.

46 Vasectomy.mp.

47 Vasovasostomy.mp.

48 or/28-47

49 Nephroureterectomy.mp.

50 Renal arterial graft*.mp.

51 Splenorenal arterial bypass.mp.

52 Hepatorenal arterial bypass.mp.

53 Pyeloplasty.mp.

54 Pyeloureteroplasty.mp.

55 Symphysiotomy of horseshoe kidney.mp.

56 Nephrostomy.mp.

57 Pyelostomy.mp.

58 Endopyelotomy.mp.

59 Pyelonephrolithotomy.mp.

60 Ureterocalicostomy.mp.

61 Ureterostomy.mp. or exp Ureterostomy/

62 Ureterolithotomy.mp.

63 Psoas hitch.mp.

64 Boari flap.mp.

65 Ureterocolic anastomosis.mp.

66 Urinary Diversion.mp. or exp Urinary Diversion/

67 Ureteroileal conduit.mp.

68 Ureteroureterostomy.mp.

69 Transureteroureterostomy.mp.

70 Ureterovaginal fistula repair.mp.

71 Repair of ureteral injuries.mp.

72 Ureterectomy.mp.

73 Ureteral reimplantation.mp.

74 Megaureter repair.mp.

75 Ureteroscopy.mp. or exp Ureteroscopy/

76 Laparoscopic surgery of the ureter.mp.

77 Retroperitoneal lymph node dissection.mp.

78 Ureterolysis for retroperitoneal fibrosis.mp.

79 Intracorporeal lithotripsy.mp.

80 Lithotripsy/ or Lithotripsy.mp.

81 Lithotripsy Laser.mp. or exp Lithotripsy, Laser/

82 ESWL.mp.

83 Extracorporeal shock wave lithotripsy.mp.

84 Excision of urachus.mp.

85 Repair of ruptured bladder.mp.

86 Vesical diverticulectomy.mp.

87 Cystolithotomy.mp.

88 Vesical diverticulectomy.mp.

89 Cystolithotomy.mp.

90 Closure of fistula.mp.

91 Transurethral resection of bladder tumor.mp.

92 TURBT.mp.

93 Laser treatment of bladder cancer.mp.

94 Augmentation cystoplasty.mp.

95 Sacral nerve stimulation.mp.

96 Vesicolithotomy.mp.

97 Meatotomy.mp.

98 Excision of stricture.mp.

99 Diverticulectomy.mp.

100 Artificial urinary sphincter implant.mp.

101 Urethroplasty.mp.

102 Urethrectomy.mp.

103 Repair of urethral fistula.mp.

104 Fistula repair.mp.

105 Dilation of stricture.mp.

106 Endoscopic treatment of urethral strictures.mp. (9)

107 Transurethral resection of the prostate.mp. or exp "Transurethral Resection of Prostate"/

108 TURP.mp.

109 Needle biopsy of the prostate.mp.

110 Prostatic stenting.mp.

111 Orchiopexy.mp. or exp Orchiopexy/

112 Hydrocelectomy.mp.

113 Varicocelectomy.mp.

114 Spermatocelectomy.mp.

115 Microscopic vasovasostomy.mp.

116 Microscopic vasoepididymostomy.mp.

117 Management of male factor infertility.mp.

118 Management of testicular torsion.mp.

119 or/49-118

120 48 or 119

121 exp Time Factors/ or (time adj2 factor*).mp. [mp=title, book title, abstract, original title, name of substance word, subject heading word, floating sub-heading word, keyword heading word, organism supplementary concept word, protocol supplementary concept word, rare disease supplementary concept word, unique identifier, synonyms]

122 (time adj2 period*).mp.

123 (time adj2 course*).mp.

124 duration*.mp.

125 prognosis.mp. or exp Prognosis/

126 Forecasting.mp. or exp Forecasting/

127 outcome prediction.mp.

128 timing.mp.

129 or/121-128

130 exp Venous Thromboembolism/ or vte.mp.

131 Venous Thromboembolism*.mp.

132 exp Venous Thrombosis/ or venous thrombosis*.mp.

133 deep vein thrombosis*.mp.

134 dvt.mp.

135 exp Pulmonary Embolism/ or pulmonary embolism*.mp.

136 exp Thromboembolism/ or thromboembolism*.mp.

137 thromboprophylaxis.mp.

138 or/130-137

139 18 or 27 or 120

Annotation: gyne OR gastro OR uro

140 129 and 138 and 139

## Appendix S2. Supplementary Methods: SCOPUS search history

( ( ( TITLE-ABS-KEY ( appendectom* OR appendicectom* OR colectomy* OR proctocolectom* OR cholecystectom* OR duodenectom* OR gastrectom* OR hernioplast* OR herniorrhaph* OR herniotom* OR jejunectom* OR pancreatectom* OR pancreaticojejunostom* OR pancreaticoduodenectom* OR duodenopancreatectom* OR hepatectom* OR splenectomy* ) ) OR ( ( ( TITLE-ABS-KEY ( ( surgery OR resection* OR excision* OR repair* ) W/3 ( abdominoperineal OR perineal OR anal* OR anus OR appendix OR bowel* OR colon* ) ) ) OR ( TITLE-ABS-KEY ( ( operation* OR laproscop* OR laparoscop* OR sleeve* ) W/3 ( abdominoperineal OR perineal OR anal* OR anus OR appendix OR bowel* OR colon* ) ) ) ) OR ( ( TITLE-ABS-KEY ( ( surgery OR resection* OR excision* OR repair* ) W/3 ( duoden* OR jejun* OR ileal* OR ileum* OR intestine* OR "gall bladder" OR gallbladder ) ) OR TITLE-ABS-KEY ( ( operation* OR laproscop* OR laparoscop* OR sleeve* ) W/3 ( duoden* OR jejun* OR ileal* OR ileum* OR intestine* OR "gall bladder" OR gallbladder ) ) ) ) OR ( ( TITLE-ABS-KEY ( ( surgery OR resection* OR excision* OR repair* ) W/3 ( gastric OR bariatric* OR stomach OR hernia OR liver OR adenoma ) ) OR TITLE-ABS-KEY ( ( operation* OR laproscop* OR laparoscop* OR sleeve* ) W/3 ( gastric OR bariatric* OR stomach OR hernia OR liver OR adenoma ) ) ) ) OR ( ( TITLE-ABS-KEY ( ( surgery OR resection* OR excision* OR repair* ) W/3 ( hepatoma* OR hepatocellular* OR rectal* OR rectum ) ) OR TITLE-ABS-KEY ( ( operation* OR laproscop* OR laparoscop* OR sleeve* ) W/3 ( hepatoma* OR hepatocellular* OR rectal* OR rectum ) ) ) ) ) OR ( ( TITLE-ABS-KEY ( ( general OR abdominal OR major ) W/3 ( surgery OR surgical ) ) OR TITLE-ABS-KEY ( prolapse W/3 rectal ) ) ) OR ( TITLE-ABS-KEY ( ( surger* OR surgic* ) W/2 ( "Digestive System" OR cholecystitis OR pancrea* OR spleen OR splenic ) ) ) OR ( TITLE-ABS-KEY ( "General Surgery" OR "digestive system surgical procedure*" ) ) OR ( TITLE-ABS-KEY ( rectopexy OR rectosigmoidectom* OR sigmoidectom* OR dhoore OR d'hoore OR delorme OR altemeier ) ) ) OR ( ( ( TITLE-ABS-KEY ( ( "Gynecologic Surgical Procedure*" OR "Oocyte Retrieval" OR "Dilatation and Curettage" OR hysteroscopy ) ) ) OR ( ( ( TITLE-ABS-KEY ( ( "Genital Diseas*" OR genitalia ) W/2 female ) ) OR ( TITLE-ABS-KEY ( "Uterine Artery" OR gynecology OR "Female Urogenital Disease*" OR "Ovarian Cyst*" OR ( pregnancy W/2 ectopic ) OR "Pregnancy Complication*" OR "Pelvic Organ Prolapse" ) ) ) AND ( TITLE-ABS-KEY ( surger* OR surgic* ) ) ) ) OR ( ( ( TITLE-ABS-KEY ( ( urethra OR "Urinary Bladder" OR "Urinary Incontinence" ) AND ( surger* OR surgic* ) ) OR TITLE-ABS-KEY ( "Suburethral Sling*" OR "Sterilization Reversal" ) ) ) AND ( TITLE-ABS-KEY ( female ) ) ) OR ( TITLE-ABS-KEY ( ( ( gyn?ecolog* OR ovar* OR vulv* OR vagin* OR transvagin* OR adnex* OR fibroid* OR myoma OR leiomyoma OR pelvic OR perineal OR endometri* OR cervic* OR cervix OR uterus OR uterin* OR fallop* OR curettage OR labia* OR clitoris ) W/3 ( resect* OR excision* OR ablation OR conisation OR conization OR endoscop* OR embol* OR operation OR torsion OR amputation OR rupture OR cytoreduc* OR repair OR fixation OR reconstruct* OR surgery OR surgeries OR procedure* OR removal ) ) OR ( cervicopex* OR cervicectom* OR colpectom* OR colporrhagh OR compopex* OR colposcop* OR colposuspen* OR colpotom* OR culdoplast* OR exenteration* OR fibroidectom* OR fimbriectom* OR hysterectom* OR hysteropex* OR hysteroscop* OR hysterosalpingo* OR lavh OR leep OR lletz OR usls OR myomectomy* OR oophorectom* OR omentectom* OR ovariectom* OR perineoplast* OR perineorrhaph* OR sacrocolpop* OR sacropex* OR salpingectom* OR salpingostom* OR salpingo-oophorectom* OR trachelectom* OR tubectom* OR tuboplast* OR uteroscop* OR vaginotom* OR vaginoplast* OR vulvectom* OR wertheim OR labiaplast* ) ) ) OR ( ( TITLE-ABS-KEY ( ( oocyte* W/2 ( retriev* OR aspirat* OR collect* ) ) ) OR TITLE-ABS-KEY ( ( tubal W/2 ( excision OR ligation OR occlusion OR ring OR rings OR sterilization OR reanastomosis ) ) ) OR TITLE-ABS-KEY ( ( ( vaginal OR pubovaginal OR transvaginal OR retropubic OR transobturator OR prolapse ) W/3 ( tape* OR sling* OR mesh* ) ) ) OR TITLE-ABS-KEY ( ( tvt OR mini-sling OR miniarc OR bulkamid ) ) OR SRCTITLE ( gyneco* W/3 surg* ) OR TITLE-ABS-KEY ( excision W/3 loop ) ) ) OR ( ( TITLE-ABS-KEY ( ( ovar* OR luteum OR luteal ) W/3 ( cystectom* OR resect* OR enucleat* OR excision* OR ablation OR conisation OR conization OR endoscop* OR embol* OR operation OR torsion OR amputation OR rupture OR cytoreduc* OR repair OR fixation OR reconstruct* OR surgery OR surgeries OR procedure* OR removal ) ) ) OR ( TITLE-ABS-KEY ( ( ( ectopic OR extrauterin* OR tubal* ) W/3 pregnan* ) AND ( cystectom* OR resect* OR enucleat* OR excision* OR ablation OR conisation OR conization OR endoscop* OR embol* OR operation OR torsion OR amputation OR rupture OR cytoreduc*or AND repair OR fixation OR reconstruct* OR surgery OR surgeries OR procedure* OR removal ) ) ) OR ( TITLE-ABS-KEY ( ( sacrospinous OR uterosacral OR "sacral ligament" ) W/3 ( fixat* OR suspens* OR plicat* ) ) ) OR ( TITLE-ABS-KEY ( ( dilatation OR curettage OR evacuation ) W/5 ( conception OR cervix OR cervic* OR uter* OR pregnancy OR placent* ) ) ) ) ) OR ( ( TITLE-ABS-KEY ( "Urologic Surgical Procedures" OR "Ambulatory Surgical Procedur*" cystectomy OR cystoscopy OR cystostomy OR cystotomy OR "Kidney Transplantation" OR nephrectomy OR ureteroscopy OR "Urinary Diversion" OR ( nephrostomy W/1 percutaneous ) ) ) OR ( TITLE-ABS-KEY ( ( circumcision W/1 male ) OR orchiectomy OR orchiopexy OR "Penile Implantation" OR prostatectomy OR vasectomy OR vasovasostomy OR nephroureterectomy OR arterial AND graft* OR "Splenorenal arterial bypass" OR "Hepatorenal arterial bypass" ) ) OR ( TITLE-ABS-KEY ( pyeloplasty OR pyeloureteroplasty OR "Symphysiotomy of horseshoe kidney" OR nephrostomy OR pyelostomy OR endopyelotomy OR pyelonephrolithotomy OR ureterocalicostomy OR ureterostomy OR ureterolithotomy OR "Psoas hitch" OR "Boari flap" ) ) OR ( TITLE-ABS-KEY ( "Ureterocolic anastomosis" OR "Urinary Diversion" OR "Ureteroileal conduit" OR ureteroureterostomy OR transureteroureterostomy OR "Ureterovaginal fistula repair" OR "Repair of ureteral injuries" OR ureterectomy OR "Ureteral reimplantation" ) ) OR ( TITLE-ABS-KEY ( "Megaureter repair" OR ureteroscopy OR "Laparoscopic surgery of the ureter" OR "Retroperitoneal lymph node dissection" OR "Ureterolysis for retroperitoneal fibrosis" OR "Intracorporeal lithotripsy" OR lithotripsy OR "Lithotripsy Laser" OR eswl ) ) OR ( TITLE-ABS-KEY ( "Extracorporeal shock wave lithotripsy" OR "Excision of urachus" OR "Repair of ruptured bladder" OR "Vesical diverticulectomy" OR cystolithotomy OR "Vesical diverticulectomy" OR cystolithotomy OR "Closure of fistula" ) ) OR ( TITLE-ABS-KEY ( "Transurethral resection of bladder tumor" OR turbt OR "Laser treatment of bladder cancer" OR "Augmentation cystoplasty" OR "Sacral nerve stimulation" OR vesicolithotomy OR meatotomy OR "Excision of stricture" OR diverticulectomy ) ) OR ( TITLE-ABS-KEY ( "Artificial urinary sphincter implant" OR urethroplasty OR urethrectomy OR "Repair of urethral fistula" OR "Fistula repair" OR "Dilation of stricture" OR "Endoscopic treatment of urethral strictures" OR "Transurethral resection of the prostate" OR turp ) ) OR ( ( TITLE-ABS-KEY ( "Needle biopsy of the prostate" OR "Prostatic stenting" OR orchiopexy OR hydrocelectomy OR varicocelectomy OR spermatocelectomy OR "Microscopic vasovasostomy" OR "Microscopic vasoepididymostomy" OR "Management of male factor infertility" ) OR TITLE-ABS-KEY ( "Management of testicular torsion" ) ) ) ) ) AND ( ( ( TITLE-ABS-KEY ( ( "Gynecologic Surgical Procedure*" OR "Oocyte Retrieval" OR "Dilatation and Curettage" OR hysteroscopy ) ) ) OR ( ( ( TITLE-ABS-KEY ( ( "Genital Diseas*" OR genitalia ) W/2 female ) ) OR ( TITLE-ABS-KEY ( "Uterine Artery" OR gynecology OR "Female Urogenital Disease*" OR "Ovarian Cyst*" OR ( pregnancy W/2 ectopic ) OR "Pregnancy Complication*" OR "Pelvic Organ Prolapse" ) ) ) AND ( TITLE-ABS-KEY ( surger* OR surgic* ) ) ) ) OR ( ( ( TITLE-ABS-KEY ( ( urethra OR "Urinary Bladder" OR "Urinary Incontinence" ) AND ( surger* OR surgic* ) ) OR TITLE-ABS-KEY ( "Suburethral Sling*" OR "Sterilization Reversal" ) ) ) AND ( TITLE-ABS-KEY ( female ) ) ) OR ( TITLE-ABS-KEY ( ( ( gyn?ecolog* OR ovar* OR vulv* OR vagin* OR transvagin* OR adnex* OR fibroid* OR myoma OR leiomyoma OR pelvic OR perineal OR endometri* OR cervic* OR cervix OR uterus OR uterin* OR fallop* OR curettage OR labia* OR clitoris ) W/3 ( resect* OR excision* OR ablation OR conisation OR conization OR endoscop* OR embol* OR operation OR torsion OR amputation OR rupture OR cytoreduc* OR repair OR fixation OR reconstruct* OR surgery OR surgeries OR procedure* OR removal ) ) OR ( cervicopex* OR cervicectom* OR colpectom* OR colporrhagh OR compopex* OR colposcop* OR colposuspen* OR colpotom* OR culdoplast* OR exenteration* OR fibroidectom* OR fimbriectom* OR hysterectom* OR hysteropex* OR hysteroscop* OR hysterosalpingo* OR lavh OR leep OR lletz OR usls OR myomectomy* OR oophorectom* OR omentectom* OR ovariectom* OR perineoplast* OR perineorrhaph* OR sacrocolpop* OR sacropex* OR salpingectom* OR salpingostom* OR salpingo-oophorectom* OR trachelectom* OR tubectom* OR tuboplast* OR uteroscop* OR vaginotom* OR vaginoplast* OR vulvectom* OR wertheim OR labiaplast* ) ) ) OR ( ( TITLE-ABS-KEY ( ( oocyte* W/2 ( retriev* OR aspirat* OR collect* ) ) ) OR TITLE-ABS-KEY ( ( tubal W/2 ( excision OR ligation OR occlusion OR ring OR rings OR sterilization OR reanastomosis ) ) ) OR TITLE-ABS-KEY ( ( ( vaginal OR pubovaginal OR transvaginal OR retropubic OR transobturator OR prolapse ) W/3 ( tape* OR sling* OR mesh* ) ) ) OR TITLE-ABS-KEY ( ( tvt OR mini-sling OR miniarc OR bulkamid ) ) OR SRCTITLE ( gyneco* W/3 surg* ) OR TITLE-ABS-KEY ( excision W/3 loop ) ) ) OR ( ( TITLE-ABS-KEY ( ( ovar* OR luteum OR luteal ) W/3 ( cystectom* OR resect* OR enucleat* OR excision* OR ablation OR conisation OR conization OR endoscop* OR embol* OR operation OR torsion OR amputation OR rupture OR cytoreduc* OR repair OR fixation OR reconstruct* OR surgery OR surgeries OR procedure* OR removal ) ) ) OR ( TITLE-ABS-KEY ( ( ( ectopic OR extrauterin* OR tubal* ) W/3 pregnan* ) AND ( cystectom* OR resect* OR enucleat* OR excision* OR ablation OR conisation OR conization OR endoscop* OR embol* OR operation OR torsion OR amputation OR rupture OR cytoreduc*or AND repair OR fixation OR reconstruct* OR surgery OR surgeries OR procedure* OR removal ) ) ) OR ( TITLE-ABS-KEY ( ( sacrospinous OR uterosacral OR "sacral ligament" ) W/3 ( fixat* OR suspens* OR plicat* ) ) ) OR ( TITLE-ABS-KEY ( ( dilatation OR curettage OR evacuation ) W/5 ( conception OR cervix OR cervic* OR uter* OR pregnancy OR placent* ) ) ) ) ) AND ( TITLE-ABS-KEY ( vte OR "Venous Thromboembolism*" OR "venous thrombosis*" OR "deep vein thrombosis*" OR dvt OR "pulmonary embolism*" OR thromboembolism* OR thromboprophylaxis ) )

## Appendix S3. Supplementary Methods: CINAHL search history

| **#** | **Query** |
| --- | --- |
| S103 | S91 AND S97 AND S102 |
| S102 | S98 OR S99 OR S100 OR S101 |
| S101 | (MH "Forecasting") |
| S100 | (MH "Prognosis+") |
| S99 | time N2 factor* OR time N2 period* OR time N2 course* OR duration* OR outcome prediction OR timing OR prognosis OR Forecasting |
| S98 | (MH "Time Factors") |
| S97 | S92 OR S93 OR S94 OR S95 OR S96 |
| S96 | (MH "Venous Thrombosis+") |
| S95 | (MH "Thromboembolism+") |
| S94 | (MH "Pulmonary Embolism") |
| S93 | vte OR Venous Thromboembolism* OR venous thrombosis* OR deep vein thrombosis* OR dvt OR pulmonary embolism* OR thromboembolism* OR thromboprophylaxis |
| S92 | (MH "Venous Thromboembolism") |
| S91 | S32 OR S69 OR S90 |
| S90 | S75 OR S89 |
| S89 | S76 OR S77 OR S78 OR S79 OR S80 OR S81 OR S82 OR S83 OR S84 OR S85 OR S86 OR S87 OR S88 |
| S88 | Nephrostomy Percutaneous OR Circumcision male OR Ureterostomy OR Urinary Diversion OR Ureteroscopy OR Lithotripsy OR Transurethral Resection of Prostate OR Ureterolysis for retroperitoneal fibrosis OR Intracorporeal lithotripsy OR Repair of ruptured bladder |
| S87 | Orchiopexy |
| S86 | (MH "Transurethral Resection of Prostate") |
| S85 | (MH "Lithotripsy+") OR (MH "Lithotripsy, Laser") |
| S84 | (MH "Ureteroscopy") |
| S83 | (MH "Urinary Diversion+") |
| S82 | (MH "Ureterostomy") |
| S81 | (MH "Circumcision") |
| S80 | (MH "Nephrostomy, Percutaneous") |
| S79 | Spermatocelectomy OR Microscopic vasovasostomy OR Microscopic vasoepididymostomy OR Management of male factor infertility OR Management of testicular torsion |
| S78 | Artificial urinary sphincter implant OR Urethroplasty OR Urethrectomy OR Repair of urethral fistula OR Fistula repair OR Dilation of stricture OR Endoscopic treatment of urethral strictures OR TURP OR Needle biopsy of the prostate OR Prostatic stenting OR Hydrocelectomy OR Varicocelectomy |
| S77 | Vesical diverticulectomy OR Closure of fistula OR Transurethral resection of bladder tumor OR TURBT OR Laser treatment of bladder cancer OR Augmentation cystoplasty OR Sacral nerve stimulation OR Vesicolithotomy OR Meatotomy OR Excision of stricture OR Diverticulectomy |
| S76 | Ureterectomy OR Ureteral reimplantation OR Megaureter repair OR Ureteroscopy OR Laparoscopic surgery of the ureter OR Retroperitoneal lymph node dissection OR Lithotripsy Laser OR ESWL OR Extracorporeal shock wave lithotripsy OR Excision of urachus OR Vesical diverticulectomy OR Cystolithotomy |
| S75 | S70 OR S71 OR S72 OR S73 OR S74 |
| S74 | Pyelonephrolithotomy OR Ureterocalicostomy OR Ureterostomy OR Ureterolithotomy OR Psoas hitch OR Boari flap OR Ureterocolic anastomosis OR Ureteroileal conduit OR Ureteroureterostom OR Transureteroureterostomy OR Ureterovaginal fistula repair OR Repair of ureteral injuries |
| S73 | Vasectomy OR Vasovasostomy OR Nephroureterectomy OR Renal arterial graft* OR Splenorenal arterial bypass OR Hepatorenal arterial bypass OR Pyeloplasty OR Pyeloureteroplasty OR Symphysiotomy of horseshoe kidney OR Nephrostomy OR Pyelostomy OR Endopyelotomy |
| S72 | Cystectomy OR Cystoscopy OR Cystostomy OR Cystotomy OR Kidney Transplantation OR Nephrectomy OR Ureteroscopy OR Urinary Diversion OR Orchiectomy OR Orchiopexy OR Penile Implantation OR Prostatectomy |
| S71 | Urologic Surgical Procedure* OR urogenital surger* |
| S70 | (MH "Surgery, Urogenital+") OR (MH "Ambulatory Surgery") |
| S69 | S61 OR S68 |
| S68 | S62 OR S63 OR S64 OR S65 OR S66 OR S67 |
| S67 | (dilatation or curettage or evacuation) N5 (conception or cervix or cervic* or uter* or pregnancy or placent*) |
| S66 | (sacrospinous or uterosacral or sacral ligament) N3 (fixat* or suspens* or plicat*) |
| S65 | (ectopic or extrauterin* or tubal*) N3 pregnan* N3 (cystectom* or resect* or enucleat* or excision* or ablation or conisation or conization or endoscop* or embol* or operation or torsion or amputation or rupture or cytoreduc*or repair or fixation or reconstruct* or surgery or surgeries or procedure* or removal) |
| S64 | (ovar* or luteum or luteal) N3 (cystectom* or resect* or enucleat* or excision* or ablation or conisation or conization or endoscop* or embol* or operation or torsion or amputation or rupture or cytoreduc*or repair or fixation or reconstruct* or surgery or surgeries or procedure* or removal) |
| S63 | excision N3 loop |
| S62 | SO Gyneco* N3 surg* |
| S61 | S58 OR S59 OR S60 |
| S60 | S43 OR S44 OR S45 OR S46 OR S47 OR S48 |
| S59 | S33 OR S34 OR S35 OR S36 OR S37 OR S38 OR S39 OR S49 OR S50 OR S51 OR S52 OR S54 OR S55 |
| S58 | S40 OR S41 OR S42 OR S53 OR S56 |
| S57 | S40 OR S41 OR S42 OR S53 OR S56 |
| S56 | (MH "Bladder/SU") |
| S55 | (MH "Pelvic Organ Prolapse+/SU") |
| S54 | (MH "Pregnancy Complications+/SU") |
| S53 | (MH "Sterilization Reversal") |
| S52 | (MH "Hysteroscopy") |
| S51 | (MH "Dilatation and Curettage") |
| S50 | (MH "Pregnancy, Ectopic+/SU") |
| S49 | (MH "Ovarian Cysts+/SU") |
| S48 | TVT or mini-sling or miniarc or bulkamid |
| S47 | (vaginal or pubovaginal or transvaginal or retropubic or transobturator or prolapse)) N3 (tape* or sling* or mesh*) |
| S46 | Tubal N2 (excision or ligation or occlusion or ring or rings or sterilization or reanastomosis) |
| S45 | oocyte* N2 (retriev* or aspirat* or collect*) |
| S44 | cervicopex* or cervicectom* or colpectom* or colporrhagh or compopex* or colposcop* or colposuspen* or colpotom* or culdoplast* or exenteration* or fibroidectom* or fimbriectom* or hysterectom* or hysteropex* or hysteroscop* or hysterosalpingo* or LAVH or LEEP or LLETZ or USLS or myomectomy* or oophorectom* or omentectom* or ovariectom* or perineoplast* or perineorrhaph* or sacrocolpop* or sacropex* or salpingectom* or salpingostom* or salpingo-oophorectom* or trachelectom* or tubectom* or tuboplast* or uteroscop* or vaginotom* or vaginoplast* or vulvectom* or Wertheim or labiaplast* |
| S43 | (gynecolog* or gynaecolog* ovar* or vulv* or vagin* or transvagin* or adnex* or fibroid* or myoma or leiomyoma or pelvic or perineal or endometri* or cervic* or cervix or uterus or uterin* or fallop* or curettage or labia* or clitoris) N3 (resect* or excision* or ablation or conisation or conization or endoscop* or embol* or operation or torsion or amputation or rupture or cytoreduc*or repair or fixation or reconstruct* or surgery or surgeries or procedure* or removal) |
| S42 | (MH "Suburethral Slings") |
| S41 | (MH "Urinary Incontinence+/SU") |
| S40 | (MH "Urethra/SU") |
| S39 | Oocyte Retrieval |
| S38 | (MH "Female Urogenital Diseases/SU") |
| S37 | (MH "Gynecology") AND (surger* OR surgic*) |
| S36 | "Uterine Arter*" AND (surger* OR surgic*) |
| S35 | (MH "Genitalia, Female+/SU") |
| S34 | (MH "Genital Diseases, Female+/SU") |
| S33 | (MH "Surgery, Gynecologic+") |
| S32 | S1 OR S2 OR S3 OR S4 OR S5 OR S6 OR S7 OR S8 OR S9 OR S10 OR S11 OR S12 OR S13 OR S14 OR S15 OR S16 OR S17 OR S18 OR S19 OR S20 OR S21 OR S22 OR S23 OR S24 OR S25 OR S26 OR S27 OR S28 OR S29 OR S30 OR S31 |
| S31 | (MH "Pancreaticojejunostomy") |
| S30 | (MH "Pancreaticoduodenectomy") |
| S29 | (MH "Pancreatectomy") |
| S28 | (MH "Hepatectomy") |
| S27 | (MH "Gastrectomy+") |
| S26 | (MH "Colectomy+") |
| S25 | (MH "Bariatric Surgery+") |
| S24 | (MH "Appendectomy") |
| S23 | (MH "Stomach Diseases+/SU") |
| S22 | (MH "Splenic Diseases+/SU") |
| S21 | (MH "Spleen/SU") |
| S20 | (MH "Pancreatic Diseases+/SU") |
| S19 | (MH "Pancreas/SU") |
| S18 | (MH "Liver Diseases+/SU") |
| S17 | (MH "Intestinal Diseases+/SU") |
| S16 | (Hernia N1 Ventral) AND (surgery or surgical)) |
| S15 | (MH "Hernia, Abdominal+/SU") |
| S14 | (MH "Gallbladder Diseases+/SU") |
| S13 | (MH "Gallbladder/SU") |
| S12 | (MH "Cholecystitis+/SU") |
| S11 | (MH "Digestive System+/SU") |
| S10 | (MH "Splenectomy") |
| S9 | (MH "Surgery, Digestive System+") |
| S8 | General Surgery |
| S7 | (MH "Herniorrhaphy") |
| S6 | (MH "Cholecystectomy+") |
| S5 | Rectopexy or rectosigmoidectom* or sigmoidectom* or DHoore or d'hoore or Delorme or Altemeier |
| S4 | prolapse N3 rectal |
| S3 | ((general or abdominal or major) N3 (surgery or surgical)) |
| S2 | ((surgery or resection* or excision* or repair* or operation* or laproscop* or laparoscop* or sleeve*) N3 (abdominoperineal or perineal or anal* or anus or appendix or bowel* or colon* or duoden* or jejun* or ileal* or ileum* or intestine* or gallbladder or gall bladder or gallbladder or gastric or bariatric* or stomach or hernia or liver or adenoma or hepatoma* or hepatocellular* or rectal* or rectum)) |
| S1 | appendectom* or appendicectom* or colectomy* or proctocolectom* or cholecystectom* or duodenectom* or gastrectom* or hernioplast* or herniorrhaph* or herniotom* or jejunectom* or pancreatectom* or pancreaticojejunostom* or pancreaticoduodenectom* or duodenopancreatectom* |

## **Table S1.** Design features used for assessment of risk of bias

| **Domain** | **Low risk of bias** | **High risk of bias** |
| --- | --- | --- |
| **Sampling and representativeness of the population** | Consecutive patient recruitment and not more than 5% of the patient population excluded | Non-consecutive patient recruitment or more than 5% of the patient population excluded |
| **Study type** | Multinational; Multicenter in one country | Single center, not single surgeon; Single surgeon |
| **Follow up of the patients** | Loss to follow-up less than 10% | Loss to follow-up unknown or at least 10% |
| **Thromboprophylaxis documentation** | The study provides thorough information on what kinds of thromboprophylaxis regimens (at least on pharmacological) were used, duration of use and proportion of patients who used thromboprophylaxis | The study does not provide thorough information on thromboprophylaxis regimen or only provides thorough information on mechanical (but not on pharmacological) thromboprophylaxis regimen. |
| **Overall risk of bias** | One or less high risk of bias domains: Overall low risk of bias  Two or more high risk of bias domains: Overall high risk of bias | |
|  |  | |

## Appendix S4. Supplementary Methods**:** Details regarding thromboprophylaxis estimation

Survey on thromboprophylaxis practice and estimation principles

For studies that did not report on use of thromboprophylaxis, we estimated thromboprophylaxis use as follows:

1. our web-based survey on thromboprophylaxis informed our decisions
2. if our survey did not include the procedure(s) performed in the study, we identified a study that reported thromboprophylaxis for the procedure(s) from the same time period and procedure.

For studies including several procedures we used the distribution of procedures and the duration of thromboprophylaxis for each procedure to calculate the median thromboprophylaxis duration for the whole study population.

We queried 32 general surgeons (from 11 different countries) and 32 gynecologic surgeons (from 8 different countries). We got 19 responses from general surgeons (from 7 countries) and 11 responses from gynecologic surgeons (from 4 countries). We presented the surgeons with numerous surgical procedures. We asked their typical thromboprophylaxis regimens/duration of thromboprophylaxis. The survey response options included: i) No prophylaxis, ii) until ambulation, iii) during hospital stay/until discharge, iv) hospital stay + 1 week after discharge, v) hospital stay + 2 weeks after discharge, vi) hospital stay + 3 weeks after discharge, and vii) hospital stay + 4 weeks after discharge.

If our survey did not include the procedure(s) performed in the included article, we used data from previously published studies to estimate length of stay for the procedure and converted survey answers to days, separately for pharmacological and mechanical prophylaxis, as follows: i) No prophylaxis = 0 days, ii) until ambulation = 1 day, iii) hospital stay = median length of stay for the procedure reported, iv) 1 week after discharge = median length of stay + 7days, v) 2 weeks after discharge = median length of stay + 14 days, vi) 3 weeks after discharge = median length of stay + 21 days, and vii) 4 weeks after discharge = median length of stay + 28 days.

Table S2 and Table S3 of this document provide details on estimated length of mechanical and pharmacological thromboprophylaxis for different studies and procedures.

## **Table S2.** Estimated duration of thromboprophylaxis for each study and surgical procedures.

| **Reference** | **Procedures in the study population** | **Estimated length of mechanical prophylaxis (days)** | **Estimated length of pharmacologic prophylaxis (days)** |
| --- | --- | --- | --- |
| Agnelli | Cancer surgery. Site of primary tumor: Urinary bladder (18.8%), Colon (15.3%), Rectum (8.8%), Uterus (8.4%), Stomach (7.5%), Prostate (5.9%), Ovary (5.9%), Kidney (3.3%) Liver (3.0%), Pancreas (2.7%), Lung (2.4%) | 8 | 14 |
| Kwon | Colorectal surgery | 2 | 11 |
| Merkow | Thyroid/parathyroid (12%),Breast (28%), Lung (2,1%) Esophagogastric (4.7%), Hepatopancreaticobiliary (15%), Colon (29%), Rectum (5.1%), Ovary/uterus (1,9%) Prostate (3%) | 1 | 6 |
| Davenport | Colorectal surgery | 2 | 11 |
| Shah | GI tract 52%, Appendectomy and Cholecystectomy 26%, Hepatobiliarypancreatic 6%, Hernia 3,5%, Genitourinary/Gynecological 5%, Biopsy 4%, Miscellaneus 2%, Lung < 1%, Spleen < 1% | 1 | 5 |
| Tzeng 2013 | Hepatectomy | 2 | 7 |
| Lavallee | Radical cystectomy | 9 | 27 |
| Tzeng 2014 | Pancreatectomy | 3 | 7 |
| VanDlac | Radical cystectomy | 9 | 27 |
| Gross | Colorectal surgery | 2 | 11 |
| Moghadamyeghaneh 2014 | Colorectal surgery | 2 | 9 |
| Kester | Total hip arthroplasty 33%, Total knee arthroplasty 61%, Partial hip arthroplasty 2.6%, Partial knee arthroplasty 4.1% | 1 | 17 |
| Martin | Esophagectomy | 0 | 10 |
| Moghadamyeghaneh 2016 | Colorectal surgery | 2 | 9 |
| Spaniolas | Bariatric surgery | 1 | 5 |
| Jordan | Nephrectomy 54.5%, Partial nephrectomy 36.9%, Nephroureterectomy 8.6% | 2 | 3 |
| McAlpine | Prostatectomy 46.5%, Nephrectomy 38.0%, Cystectomy 10.1%, Nephroureterectomy 3.6%, Retroperitoneal lymphnode dissesction 1.­­7% | 2 | 5 |
| Benlice | Elective abdominopelvic bowel surgery | 1 | 6 |
| Herforth | Hernioplasty (11.5%), Biliary tract (11.3%), Appendix (8.2%), Colorectal (10.7%), Mastetctomy (4.9%), Bariatric (3.5%), Hernia repair (2.3%), Stomach (1.6%), Prostate (2.7%), Kidney (1.4%), Other surgery (not reported 32.6%) | 1 | 3 |
| Sager | Rotator cuff repair | 0 | 0 |
| Merhe | Prostatectomy | 1 | 1 |
| Kumar | Ventral hernia repair | 0 | 4 |

## **Table S3.** Estimated thromboprophylaxis duration for each procedure.

| **Procedure/patient recruitment years** | **Estimated length of mechanical thrombo-prophylaxis (days)** | **Estimate based on** | **Estimated length of pharma-cologic thrombo-prophylaxis (days)** | **Estimate based on** |
| --- | --- | --- | --- | --- |
| Lap appendectomy - 2011-2021 | 0 | Survey | 1 | Survey |
| Lap appendectomy - 2000-2010 | 0 | Survey | 1 | Survey |
| Open appendectomy - 2011-2021 | 0 | Survey | 2 | Survey |
| Open appendectomy - 2000-2010 | 0 | Survey | 2 | Survey |
| Lap cholecystectomy - 2011-2021 | 0 | Survey | 2 | Survey/Gundogdu 2017^1^ |
| Lap cholecystectomy - 2000-2010 | 0 | Survey | 3 | Survey |
| Open cholecystectomy - 2011-2021 | 0 | Survey | 6 | Survey |
| Open cholecystectomy - 2000-2010 | 0 | Survey | 6 | Survey |
| Lap hernia repair (groin) - 2011-2021 | 0 | Survey | 2 | Survey |
| Lap hernia repair (groin) - 2000-2010 | 0 | Survey | 2 | Survey |
| Open hernia repair (groin) - 2011-2021 | 0 | Survey | 2 | Survey |
| Open hernia repair (groin) - 2000-2010 | 0 | Survey | 2 | Survey |
| Lap hernia repair (ventral) - 2011-2021 | 0 | Survey | 2 | Survey |
| Lap hernia repair (ventral) - 2000-2010 | 0 | Survey | 2 | Survey |
| Open hernia repair (ventral) - 2011-2021 | 0 | Survey | 5 | Kraft 2019^2^/Survey |
| Open hernia repair (ventral) - 2000-2010 | 0 | Survey | 5 | Survey |
| Lap small bowel resection - 2011-2021 | 1 | Survey | 6 | Survey |
| Lap small bowel resection - 2000-2010 | 1 | Survey | 5 | Survey |
| Open small bowel resection - 2011-2021 | 1 | Survey | 6 | Survey |
| Open small bowel resection - 2000-2010 | 1 | Survey | 5 | Survey |
| Lap splenectomy (elective) - 2011-2021 | 1 | Tastaldi 2019^3^/Survey | 2 | Tastaldi 2019^3^/Survey |
| Lap splenectomy (elective) - 2000-2010 | 2 | Tastaldi 2019^3^/Survey | 2 | Tastaldi 2019^3^/Survey |
| Open splenectomy (elective) - 2011-2021 | 2 | Survey | 9 | Survey |
| Open splenectomy (elective) - 2000-2010 | 2 | Survey | 8 | Survey |
| Lap colectomy - 2011-2021 | 2 | Survey | 7 | Mukkamala 2020^4^ |
| Lap colectomy - 2000-2010 | 2 | Survey | 11 | Survey |
| Open colectomy - 2011-2021 | 2 | Survey | 9 | Mukkamala 2020^4^ |
| Open colectomy - 2000-2010 | 2 | Survey | 12 | Survey |
| Rob colectomy - 2011-2021 | 2 | Survey | 5 | Mukkamala 2020^4^ |
| Rob colectomy - 2000-2010 | 2 | Survey | 6 | Survey |
| Lap anterior resection - 2011-2021 | 1 | Survey | 6 | Mukkamala 2020^4^ |
| Lap anterior resection - 2000-2010 | 1 | Survey | 9 | Survey |
| Open anterior resection - 2011-2021 | 2 | Survey | 8 | Mukkamala 2020^4^ |
| Open anterior resection - 2000-2010 | 2 | Survey | 11 | Survey |
| Lap proctocolectomy - 2011-2021 | 1 | Survey | 7 | Mukkamala 2020^4^ |
| Lap proctocolectomy - 2000-2010 | 15 | Gu 2016^5^ | 15 | Gu 2016^5^ |
| Open proctocolectomy - 2011-2021 | 2 | Survey | 9 | Mukkamala 2020^4^ |
| Open proctocolectomy - 2000-2010 | 15 | Gu 2016^5^ | 15 | Gu 2016^5^ |
| Lap abdominoperineal resection - 2011-2021 | 2 | Survey | 8 | Mukkamala 2020^4^ |
| Open abdominoperineal resection - 2011-2021 | 2 | Survey | 11 | Mukkamala 2020^4^ |
| Lap rectopexy - 2011-2021 | 1 | Survey | 7 | Mukkamala 2020^4^ |
| Lap rectopexy - 2000-2010 | 1 | Survey | 5 | Survey |
| Open rectopexy - 2011-2021 | 1 | Survey | 9 | Mukkamala 2020^4^ |
| Open rectopexy - 2000-2010 | 1 | Survey | 6 | Survey |
| Lap gastrectomy - 2011-2021 | 1 | Survey | 14 | Survey |
| Lap gastrectomy - 2000-2010 | 1 | Survey | 6 | Survey |
| Open gastrectomy - 2011-2021 | 3 | Survey | 15 | Survey |
| Open gastrectomy - 2000-2010 | 2 | Survey | 7 | Survey |
| Rob gastrectomy - 2011-2021 | 4 | Survey | 12 | Survey |
| Lap gastric bypass - 2011-2021 | 1 | Survey | 4 | Survey |
| Lap gastric bypass - 2000-2010 | 2 | Kothari 2007^6^ | 2 | Kothari 2007^6^ |
| Open gastric bypass - 2011-2021 | 1 | Survey | 6 | Survey |
| Open gastric bypass - 2000-2010 | 2 | Caruana 2009^7^, Cotter 2005^8^ | 6 | Caruana 2009^7^, Cotter 2005^8^ |
| Rob gastric bypass - 2011-2021 | 1 | Survey | 4 | Survey |
| Rob gastric bypass - 2000-2010 | 1 | Survey | 3 | Survey |
| Lap sleeve gastrectomy - 2011-2021 | 1 | Nimeri 2018^9^, Moradian 2017^10^ | 1 | Nimeri 2018^9^, Moradian 2017^10^ |
| Lap sleeve gastrectomy - 2000-2010 | 1 | Survey | 2 | Survey |
| Rob sleeve gastrectomy - 2011-2021 | 1 | Survey | 2 | Survey |
| Lap liver resection - 2011-2021 | 1 | Survey | 10 | Ruff 2019^11^, Weiss 2014^12^ |
| Lap liver resection - 2000-2010 | 1 | Survey | 4 | Survey |
| Open liver resection - 2011-2021 | 2 | Survey | 15 | Ruff 2019^11^, Weiss 2014^12^ |
| Open liver resection - 2000-2010 | 2 | Survey | 10 | Barbas 2013^13^ |
| Lap pancreaticoduodenectomy - 2011-2021 | 3 | Survey | 11 | Ruff 2019^11^, Weiss 2014^12^ |
| Lap pancreaticoduodenectomy - 2000-2010 | 3 | Survey | 7 | Kendrick 2010^14^ |
| Open pancreaticoduodenectomy - 2011 2021 | 2 | Survey | 14 | Ruff 2019^11^, Weiss 2014^12^ |
| Open pancreaticoduodenectomy - 2000-2010 | 3 | Survey | 13 | Survey |
| Rob pancreaticoduodenectomy - 2011-2021 | 1 | Survey | 10 | Ruff 2019^11^, Weiss 2014^12^ |
| Open distal pancreatectomy - 2011-2021 | 2 | Survey | 11 | Ruff 2019^11^, Weiss 2014^12^ |
| Open distal pancreatectomy - 2000-2010 | 6 | Dedania 2013^15^ | 6 | Survey |
| Retroperitoneal lymphnode dissection - 2000- | 9 | Indirect evidence from radical cystectomy | 27 | Indirect evidence from radical cystectomy |
| Nephrectomy - 2000- | 2 | Hayn 2010^16^,  Reifsnyder 2012^17^,  Montgomery 2005^18^,  Kara 2015^19^ | 3 | Hayn 2010^16^,  Reifsnyder 2012^17^,  Montgomery 2005^18^,  Kara 2015^19^ |
| Prostatectomy -2000-Europe | 6 | Beyer 2019^20^,  Clément 2011^21^,  Novara 2010^22^ | 15 | Beyer 2019^20^,  Clément 2011^21^,  Novara 2010^22^ |
| Nephroureterectomy - 2000- | 2 | Indirect evidence from nephrectomy | 3 | Indirect evidence from nephrectomy |
| Prostatectomy -2000- USA | 1 | Touijer 2008^23^,  Agarwal 2011^24^,  Patel 2011^25^,  Chalmers 2013^26^,  Lasser 2010^27^ | 1 | Touijer 2008^23^,  Agarwal 2011^24^,  Patel 2011^25^,  Chalmers 2013^26^,  Lasser 2010^27^ |
| Radical /Partial cystectomy - 2000- | 9 | Shabsigh 2008^28^,  Svatek 2010^29^,  Ng 2010^30^,  Pycha 2008^31^,  Novara 2009^32^,  Clément 2011^21^,  Tilki 2010^33^ | 27 | Shabsigh 2008^28^,  Svatek 2010^29^,  Ng 2010^30^,  Pycha 2008^31^,  Novara 2009^32^,  Clément 2011^21^,  Tilki 2010^33^ |
| Esophagectomy - 2000- |  | Voeten 2020^34^,  Zwischenberger 2015^35^ |  | Voeten 2020^34^,  Zwischenberger 2015^35^ |
| Rotator cuff repair - 2010-2021 | 0 | Schick 2014^36^ | 0 | Schick 2014^36^ |
| Total/partial hip arthroplasty | 1 | Farfan 2016^37^  Flevas 2018^38^ | 17 | Farfan 2016^37^  Flevas 2018^38^ |
| Total/partial knee arthroplasty | 1 | Farfan 2016^37^  Flevas 2018^38^ | 17 | Farfan 2016^37^  Flevas 2018^38^ |

#

# Supplementary Results

## **Table S4.** Characteristics of the included studies, including information on included surgical procedures.

| **First author** | **Year** | **Surgical category; procedure** | **No. of patients** | **Female** | **Age** | **No. of VTE events** | **Recruitment years** |
| --- | --- | --- | --- | --- | --- | --- | --- |
| Agnelli | 2006 | Mixed | 2373 | 46 % | 64 | 92 | NR |
| Kwon | 2011 | General; Colorectal | 4195 | 54 % | 61 | 47 | 2005-2009 |
| Merkow | 2011 | Mixed | 44 656 | 65 % | NR | 719 | 2006-2008 |
| Davenport | 2012 | General; Colorectal cancer resection | 21 943 | 49 % | 66 | 446 | 2005-2009 |
| Shah | 2013 | Mixed | 471 867 | 41 % | 54 | 7078 | 2005-2010 |
| Tzeng | 2013 | General; Hepatectomy | 7621 | 52 % | 60* | 210 | 2005-2010 |
| Tzeng | 2014 | General; Pancreatectomy | 13 771 | 52 % | 64* | 427 | 2005-2010 |
| Lavallee | 2014 | Urologic; Radical cystectomy | 2303 | 21 % | 68 | 123 | 2006-2012 |
| VanDlac | 2014 | Urologic; Radical cystectomy | 1307 | 24 % | 69* | 78 | 2005-2011 |
| Gross | 2014 | General; Colorectal | 37 076 | 48 % | 66 | 1018 | 2005-2010 |
| Moghadamyeghaneh | 2014 | General; Colorectal | 116 029 | 52 % | 62 | 4556 | 2005-2011 |
| Kester | 2014 | Orthopedic; Knee and hip arthroplasty | 23 620 | 61 % | NR | 366 | 2008-2010 |
| Martin | 2015 | General; Esophagectomy | 3208 | NR | 64* | 161 | 2005-2012 |
| Moghadamyeghaneh | 2016 | General; Colorectal | 219 477 | 52 % | 61 | 2278 | 2005-2013 |
| Spaniolas | 2016 | General; Bariatric | 71 694 | 79 % | 45* | 283 | 2006-2011 |
| Jordan | 2017 | Urologic; Nephrectomy/NU | 13 208 | 42 % | 61 | 160 | 2006-2012 |
| McAlpine | 2017 | Urologic; Various cancer | 65 100 | NR | NR | 956 | 2006-2014 |
| Benlice | 2018 | General; Elective abdominopelvic bowel | 24 182 | 49 % | 43 | 614 | 2005-2016 |
| Herforth | 2019 | General and Urologic; Mixed | 503 602 | 51 % | NR | 3912 | 2016 |
| Sager | 2019 | Orthopedic; Rotator cuff | 39 825 | 42 % | 59 | 102 | 2005-2017 |
| Merhe | 2020 | Urologic; Prostatectomy | 36 753 | 0 % | 62 | 423 | 2008-2015 |
| Kumar | 2021 | General; Ventral Hernia | 141 065 | 57 % | 57 | 878 | 2011-2017 |
| **Total** |  |  | **1 864 875** |  |  | **24 927** |  |

NR, not reported; NU, nephroureterectomy;* Age is median if asterisk; otherwise mean

## **Table S5**. Proportion of cumulative occurrence of venous thromboembolism by time (days) during the first 28 days (4 weeks) post-surgery (all included studies pooled)

| Day | Unweighted cumulative risk (%) | Weighted cumulative risk (%) |
| --- | --- | --- |
| 1 | 4.73992747 | 4.57718270 |
| 2 | 11.32185514 | 10.32091082 |
| 3 | 18.55965368 | 17.64689496 |
| 4 | 26.40019261 | 26.37844086 |
| 5 | 34.58898720 | 35.04071251 |
| 6 | 42.41020051 | 41.41218104 |
| 7 | 49.62704252 | 47.11359695 |
| 8 | 56.04900601 | 52.30295119 |
| 9 | 61.90097009 | 57.12826723 |
| 10 | 65.77146413 | 61.03385240 |
| 11 | 69.20794674 | 64.68096183 |
| 12 | 72.26073527 | 67.94861159 |
| 13 | 75.16904797 | 71.06375774 |
| 14 | 77.92827112 | 74.02947628 |
| 15 | 80.46270606 | 76.82914131 |
| 16 | 82.81736135 | 79.45862811 |
| 17 | 84.98662200 | 81.91018264 |
| 18 | 86.90901568 | 84.14775443 |
| 19 | 88.66315936 | 86.21366728 |
| 20 | 90.26451429 | 88.11476369 |
| 21 | 91.73450423 | 89.86962320 |
| 22 | 93.09805050 | 91.49480827 |
| 23 | 94.38158056 | 93.02241498 |
| 24 | 95.60691967 | 94.48106105 |
| 25 | 96.79031957 | 95.89391204 |
| 26 | 97.94340227 | 97.27834137 |
| 27 | 99.07388246 | 98.64646932 |
| 28 | 100.00000000 | 100.00000000 |

## **Figure S1.** Proportion of cumulative occurrence of venous thromboembolism by time (days) during the first 28 days (4 weeks) post-surgery in individual studies (landscape version).

**
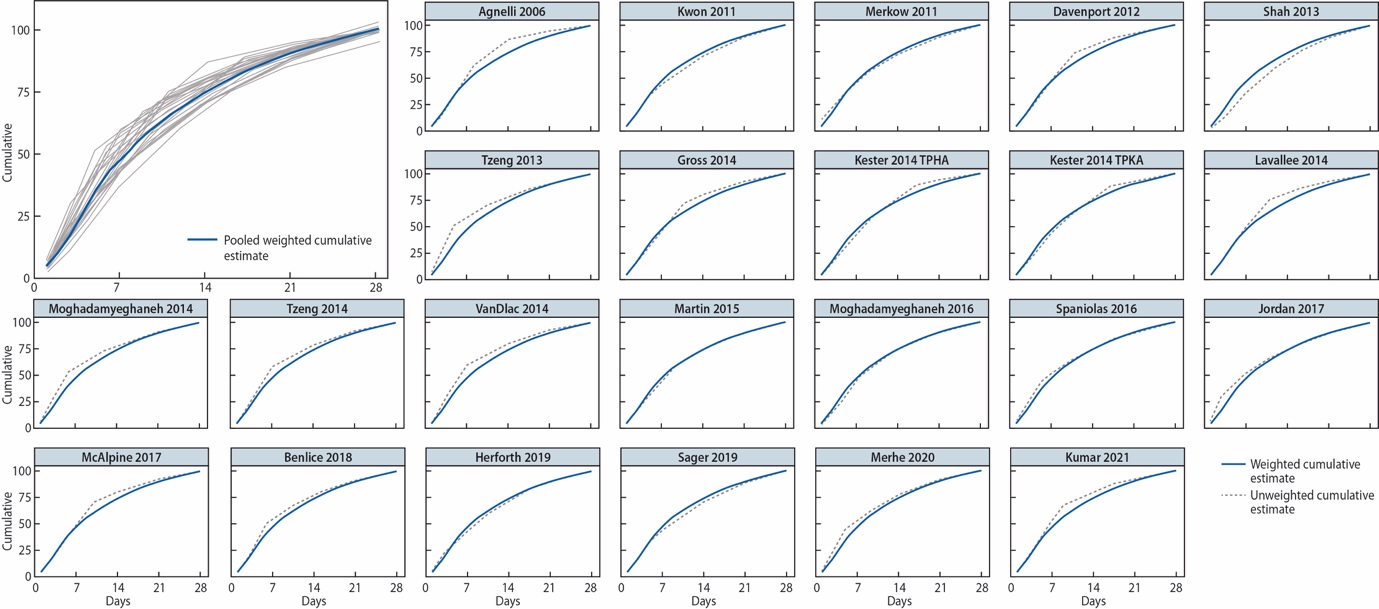
**

## Studies reporting separately pulmonary embolism (PE) and/or deep vein thrombosis (DVT) events

## **Figure S2.** Proportion of cumulative occurrence of venous thromboembolism by time (days) during the first 28 days (4 weeks) post-surgery, modelled using PE events only.


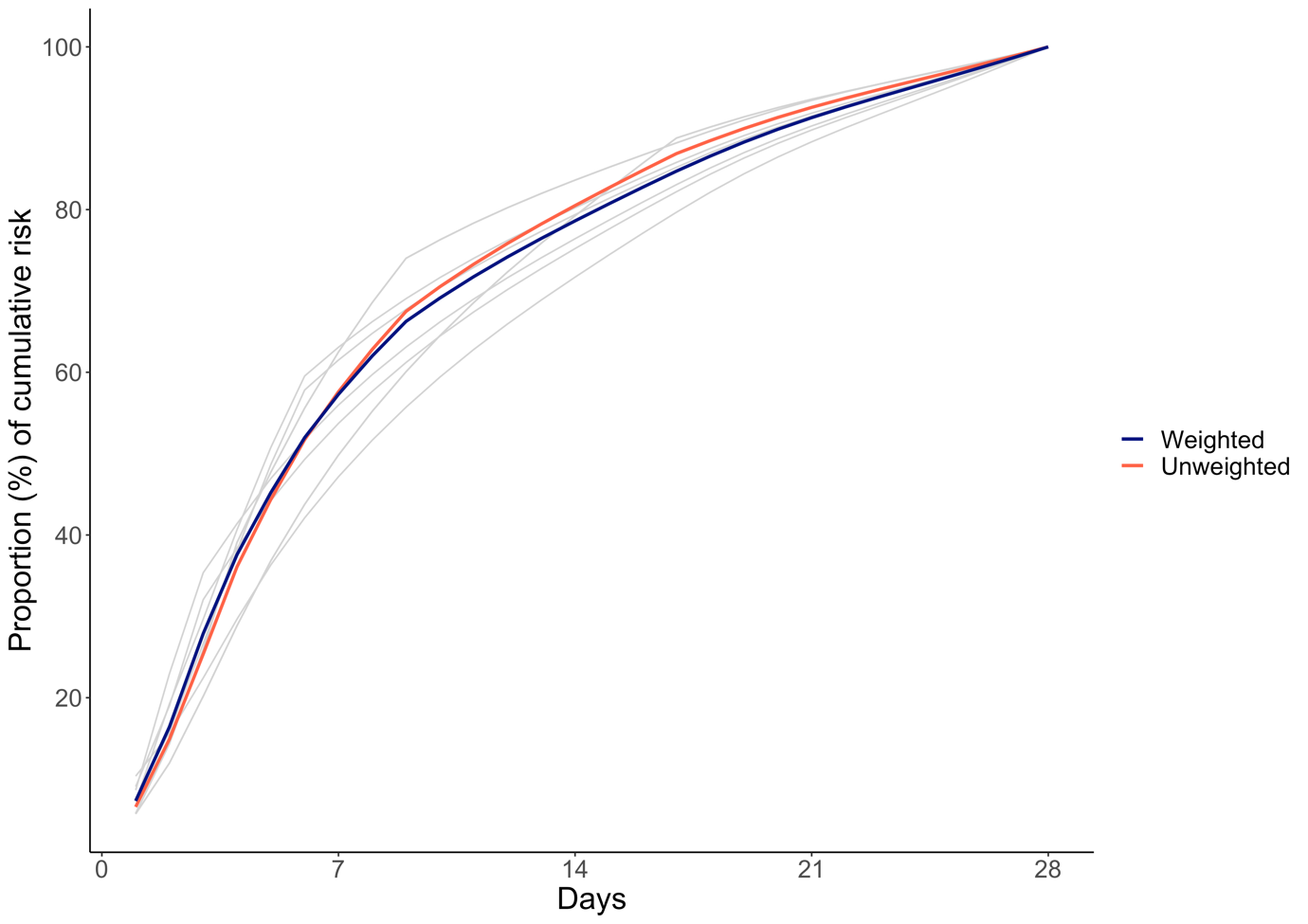


Following 8 studies reported separately PE events (n=13 152) and were included in the Figure S5: Merkow 2011, Kester 2014, Moghadamyeghaneh 2014, Moghadamyeghaneh 2016, Spaniolas 2016, Jordan 2017, Herforth 2019, Merhe 2021. Full citation information of the studies is available in the full article.

## **Figure S3**. Proportion of cumulative occurrence of venous thromboembolism by time (days) during the first 28 days (4 weeks) post-surgery, modelled using DVT events only.


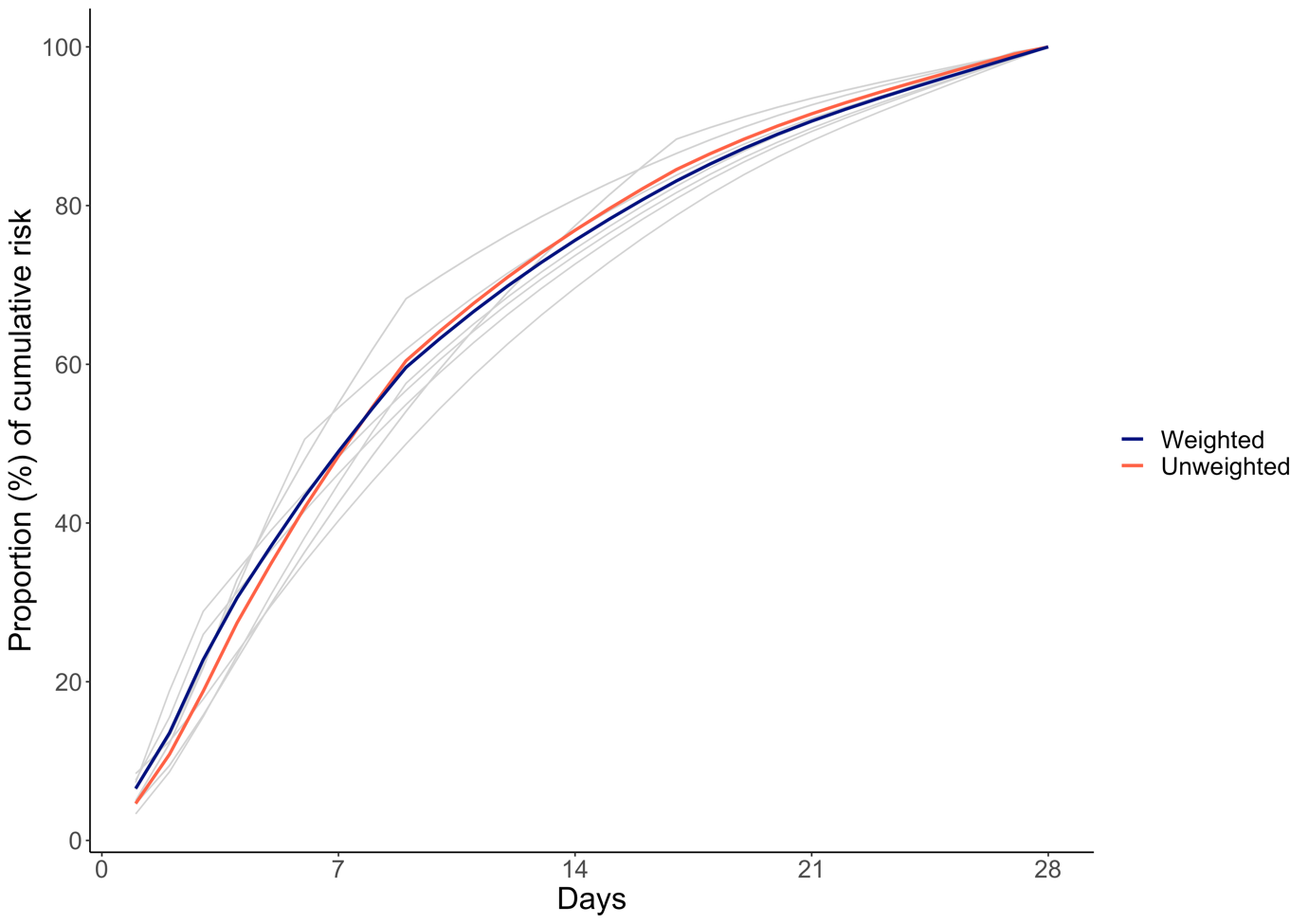


Following 8 studies reported separately DVT events (n=12 537) and were included in the Figure S6: Merkow 2011, Kester 2014, Lavallee 2014, Moghadamyeghaneh 2014, Moghadamyeghaneh 2016, Jordan 2017, Herforth 2019, Merhe 2021. Full citation information of the studies is available in the full article.

## **Figure S4.** Proportion of cumulative occurrence of venous thromboembolism by time (days) during the first 28 days (4 weeks) post-surgery (using only studies that reported on VTE events).


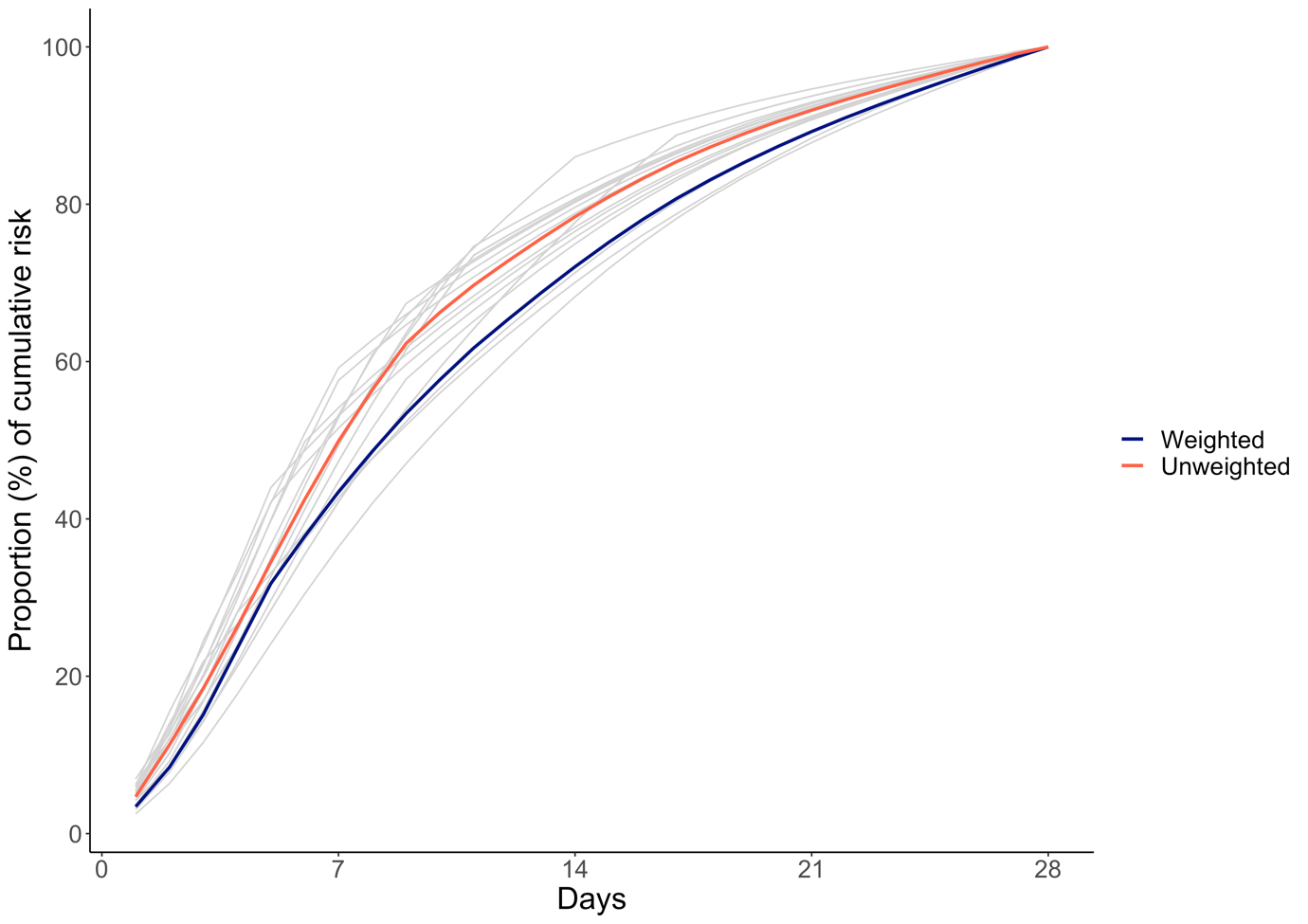


## Sensitivity analyses regarding duration of thromboprophylaxis

## **Figure S5.** Proportion of cumulative occurrence of venous thromboembolism by time (days) since surgery during the first 28 days (4 weeks) post-surgery with 0, 1, 2 and 3 weeks of estimated thromboprophylaxis (including mechanical thromboprohylaxis).

All models include 2 days of mechanical thromboprohylaxis. In the figure, wk refers to week(s) and TPX to thromboprophylaxis.


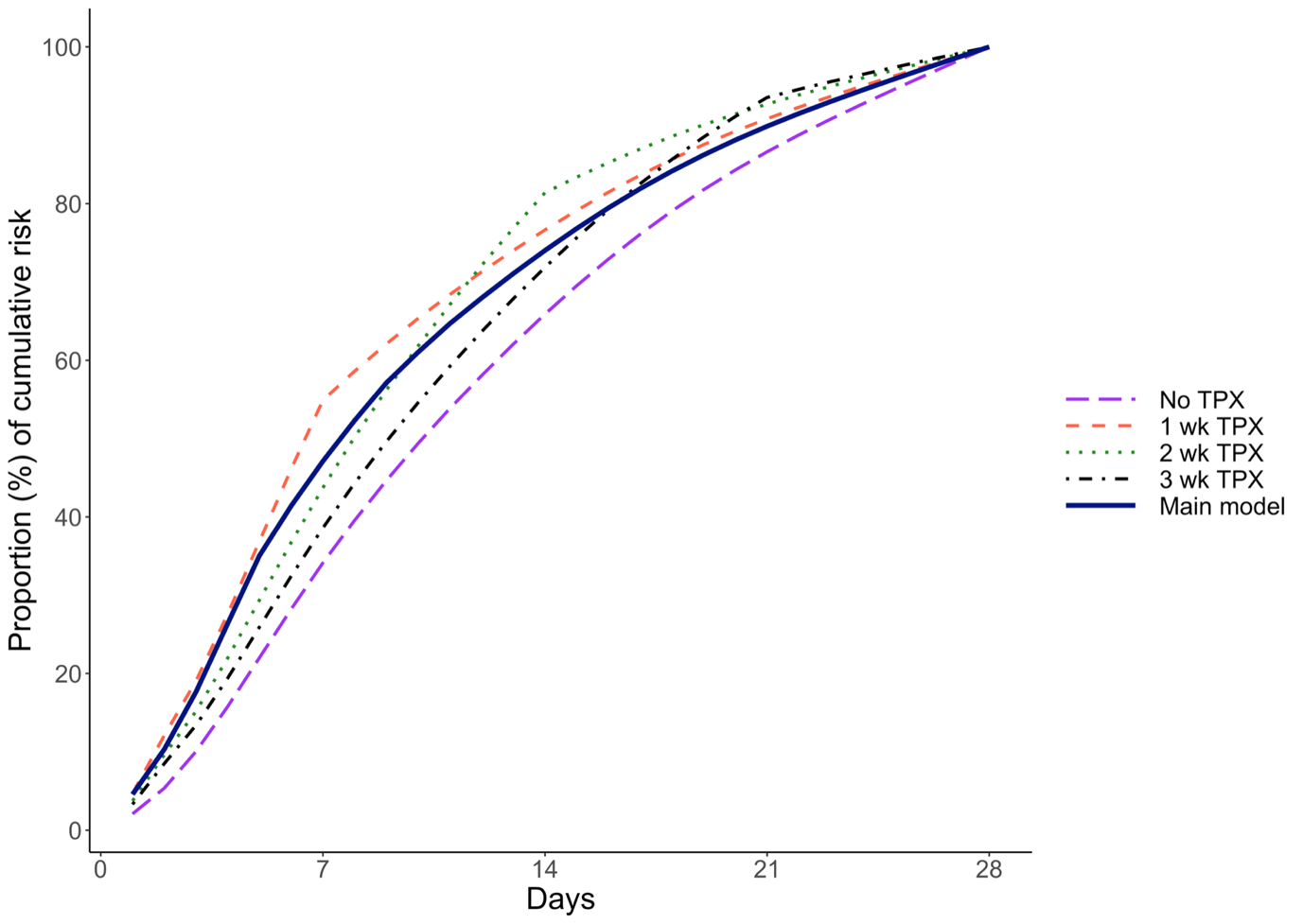


## **Figure S6.** Proportion of cumulative occurrence of venous thromboembolism by time (days) since surgery during the first 28 days (4 weeks) post-surgery with 0, 1, 2 and 3 weeks of estimated thromboprophylaxis (including mechanical thromboprohylaxis).

Only the main model includes 2 days of mechanical thromboprohylaxis. In the figure, wk refers to week(s) and TPX to thromboprophylaxis.


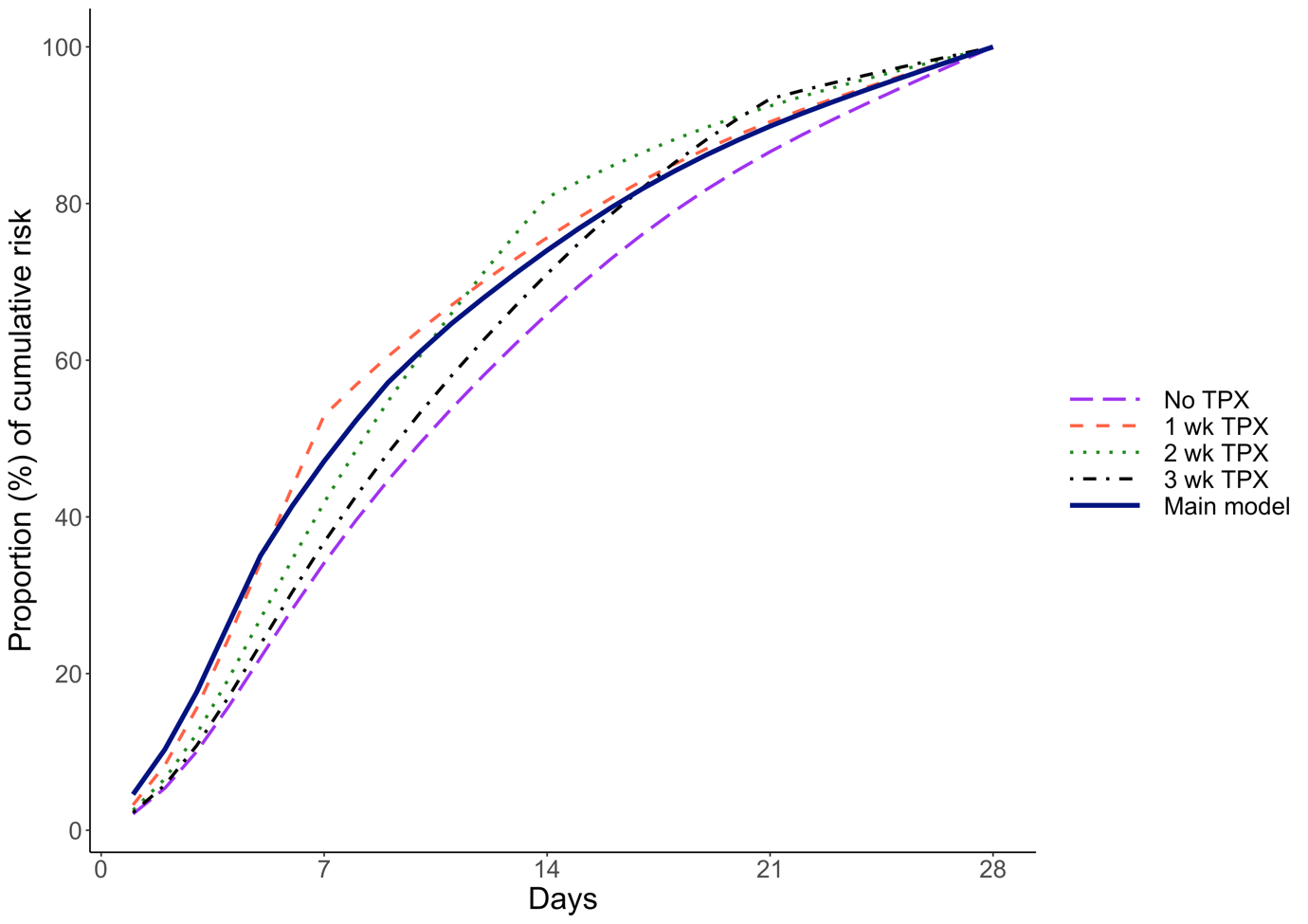


## **Figure S7.** Proportion of cumulative occurrence of venous thromboembolism (main model), pulmonary embolism (PE) and deep vein thrombosis (DVT) by time (days) during the first 28 days (4 weeks) post-surgery.


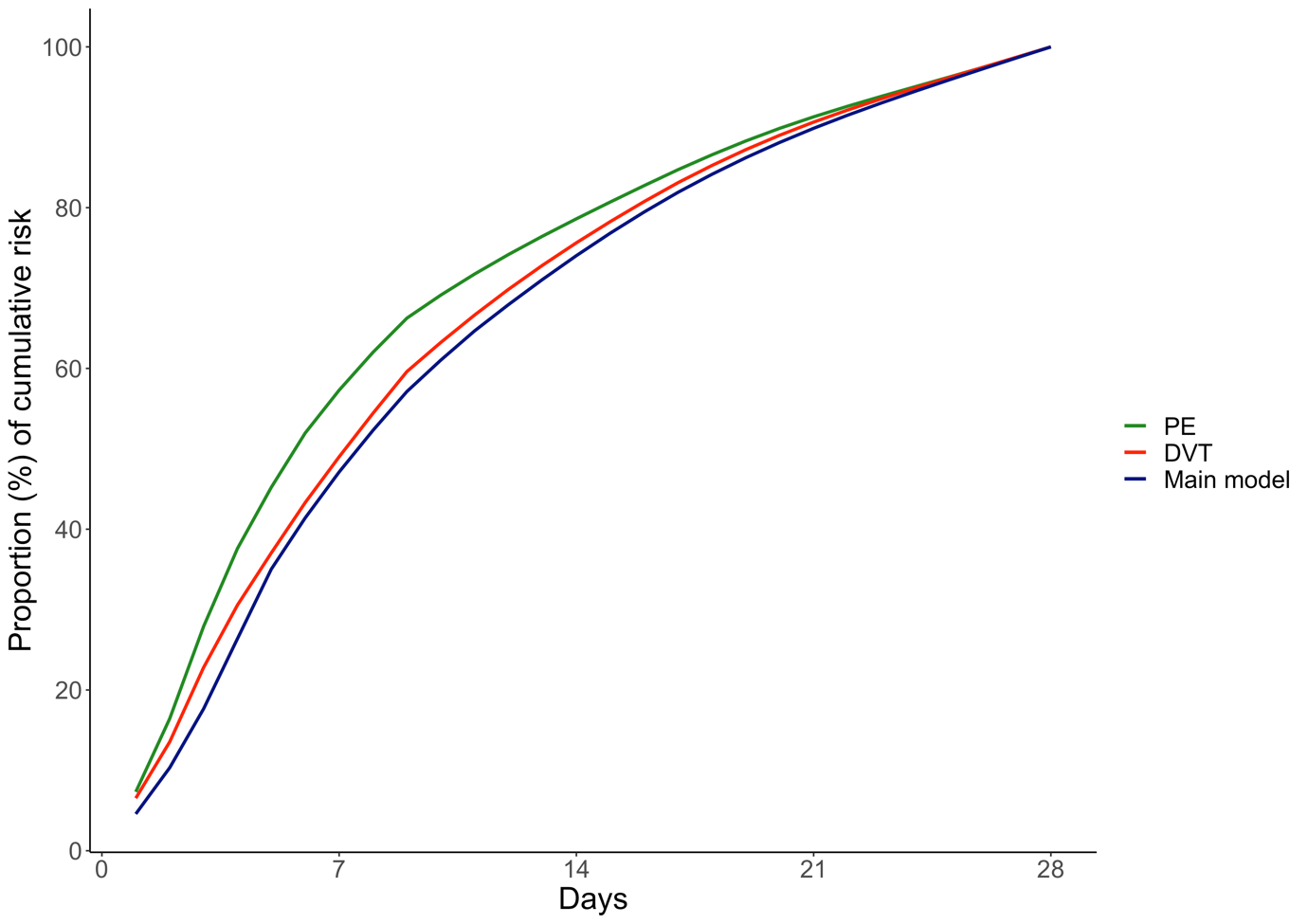


# References

## Table S3 references

1. Gundogdu RH, Oduncu M, Bozkirli BO, et al. Does thromboprophylaxis cause bleeding after laparoscopic cholecystectomy? Bratisl Lek Listy 2017;118(3):156-9.
2. Kraft CT, Janis JE. Venous hromboembolism after abdominal wall reconstruction: A prospective analysis and review of the literature. Plast Reconstr Surg 2019;15:15.
3. Tastaldi L, Krpata DM, Prabhu AS, et al. Laparoscopic splenectomy for immune thrombocytopenia (ITP): long-term outcomes of a modern cohort. Surg Endosc 2019;33(2):475-85.
4. Mukkamala A, Montgomery JR, De Roo AC, et al. Population-Based analysis of adherence to postdischarge extended venous thromboembolism prophylaxis after colorectal resection. Dis Colon Rectum 2020 Jul;63(7):911-917.
5. Gu J, Stocchi L, Gorgun E, et al. Risk factors associated with portomesenteric venous thrombosis in patients undergoing restorative proctocolectomy for medically refractory ulcerative colitis. Colorectal Dis 2016;18(4):393-9.
6. Kothari SN, Lambert PJ, Mathiason MA. A comparison of thromboembolic and bleeding events following laparoscopic gastric bypass in patients treated with prophylactic regimens of unfractionated heparin or enoxaparin. Am J Surg 2007;194(6):709-11.
7. Caruana JA, Anain PM, Pham DT. The pulmonary embolism risk score system reduces the incidence and mortality of pulmonary embolism after gastric bypass. Surgery 2009;146(4):678-83; discussion 83-5.
8. Cotter SA, Cantrell W, Fisher B, et al. Efficacy of venous thromboembolism prophylaxis in morbidly obese patients undergoing gastric bypass surgery. Obes Surg 2005;15(9):1316-20.
9. Moradian S, Daneshpajouh A, Patel A, et al. Laparoscopic sleeve gastrectomy without over-sewing the staple line: A case series demonstrating efficacy and minimization of both intra- and post-operative complications. Int J Surg Open 2017;8:7-10.
10. Nimeri AA, Bautista J, Ibrahim M, et al. Mandatory risk assessment reduces venous thromboembolism in bariatric surgery Patients. Obes Surg 2018;28(2):541-7.
11. Ruff SM, Ayabe RI, Wach MM, Diggs LP, et al. Practice patterns of VTE chemoprophylaxis after discharge following hepatic and pancreatic resections for cancer: A survey of hepatopancreatobiliary surgeons. J Thromb Thrombolysis 2019 Jul;48(1):119-124.
12. Weiss MJ, Kim Y, Ejaz A, Spolverato G, et al. Venous thromboembolic prophylaxis after a hepatic resection: patterns of care among liver surgeons. HPB (Oxford). 2014 Oct;16(10):892-8.
13. Barbas AS, Turley RS, Mallipeddi MK, et al. Examining reoperation and readmission after hepatic surgery. JACS 2013;216(5):915-23.
14. Kendrick ML, Cusati D. Total laparoscopic pancreaticoduodenectomy: feasibility and outcome in an early experience. Arch Surg. 2010;145(1):19-23.
15. Dedania N, Agrawal N, Winter JM, et al. Splenic vein thrombosis is associated with an increase in pancreas-specific complications and reduced survival in patients undergoing distal pancreatectomy for pancreatic exocrine cancer. J Gastrointest Surg 2013;17(8):1392-8.
16. Hayn MH, Schwaab T, Underwood W, Kim HL. RENAL nephrometry score predicts surgical outcomes of laparoscopic partial nephrectomy. BJU Int 2011; 108: 876-81.
17. Reifsnyder JE, Ramasamy R, Ng CK, et al. Laparoscopic and open partial nephrectomy: complication comparison using the Clavien system. JSLS 2012; 16: 38-44.
18. Montgomery JS, Wolf JS Jr. Venous thrombosis prophylaxis for urological laparoscopy: fractionated heparin versus sequential compression devices. J Urol 2005; 173: 1623-6.
19. Kara O, Zargar H, Akca O, et al. Risks and benefits of pharmacological prophylaxis for venous thromboembolism prevention in patients undergoing robotic partial nephrectomy. J Urol 2015; 195: 1348-1353.
20. Beyer J, Wessela S, Hakenberg OW, et al. Incidence, risk profile and morphological pattern of venous thromboembolism after prostate cancer surgery. J Thromb Haemost 2009; 7: 597-604.
21. Clément C1, Rossi P, Aissi K, et al. Incidence, risk profile and morphological pattern of lower extremity venous thromboembolism after urological cancer surgery. J Urol 2011; 186: 2293-7.
22. Novara G, Ficarra V, D'Elia C, Secco S, Cavalleri S, Artibani W. Prospective evaluation with standardised criteria for postoperative complications after robotic-assisted laparoscopic radical prostatectomy. Eur Urol 2010; 57: 363- 70.
23. Touijer K, Eastham JA, Secin FP, et al. Comprehensive prospective comparative analysis of outcomes between open and laparoscopic radical prostatectomy conducted in 2003 to 2005. J Urol 2008; 179: 1811-7.
24. Agarwal PK, Sammon J, Bhandari A, et al. Safety profile of robot-assisted radical prostatectomy: a standardized report of complications in 3317 patients. Eur Urol 2011; 59: 684-98.
25. Patel T, Kirby W, Hruby G, Benson MC, McKiernan JM, Badani K. Heparin prophylaxis and the risk of venous thromboembolism after robotic-assisted laparoscopic prostatectomy. BJU Int 2011; 108: 729-32.
26. Chalmers DJ1, Scarpato KR, Staff I, et al. Does heparin prophylaxis reduce the risk of venous thromboembolism in patients undergoing robot-assisted prostatectomy? J Endourol 2013; 27: 800-3.
27. Lasser MS, Renzulli J 2nd, Turini GA 3rd, Haleblian G, Sax HC, Pareek G. An unbiased prospective report of perioperative complications of robot-assisted laparoscopic radical prostatectomy. Urology 2010; 75: 1083-9.
28. Shabsigh A, Korets R, Vora KC, et al. Defining early morbidity of radical cystectomy for patients with bladder cancer using a standardized reporting methodology. Eur Urol 2009; 55: 164-74. Epub 2008 Jul 18.
29. Svatek RS, Fisher MB, Matin SF, et al. Risk factor analysis in a contemporary cystectomy cohort using standardized reporting methodology and adverse event criteria. J Urol 2010; 183: 929-34.
30. Ng CK, Kauffman EC, Lee MM, et al. A comparison of postoperative complications in open versus robotic cystectomy. Eur Urol 2010; 57: 274-81.
31. Pycha A, Comploj E, Martini T, et al. Comparison of complications in three incontinent urinary diversions. Eur Urol 2008; 54: 825-32.
32. Novara G, De Marco V, Aragona M, et al. Complications and mortality after radical cystectomy for bladder transitional cell cancer. J Urol 2009; 182: 914-21.
33. Tilki D, Zaak D, Trottmann M, et al. Radical cystectomy in the elderly patient: a contemporary comparison of perioperative complications in a single institution series. World J Urol 2010; 28: 445-50.
34. Voeten DM, van der Werf LR, van Sandick JW, van Hillegersberg R, van Berge Henegouwen MI. Length of hospital stay after uncomplicated esophagectomy. Hospital variation shows room for nationwide improvement. Surg Endosc 2021;35:6344-6357.
35. Zwischenberger BA, Tzeng CWD, Ward, ND, Zwischenberger JB, Martin JT. Venous thromboembolism prophylaxis for esophagectomy: A survey of practice patterns among thoracic surgeons. Ann Thorac Surg 2016;101:489-94
36. Schick CW, Westermann RW, Gao Y, wolf BR. Thromboembolism following shoulder arthroscopy. Orthop J Sports Med. 2014 Nov; 2(11): 2325967114559506.
37. Farfan M, Bautista M, Bonilla G, Rojas J, Llina ́s A, Navas J. Worldwide adherence to ACCP guidelines for thromboprophylaxis after major orthopedic surgery: A systematic review of the literature and meta-analysis. Thromb Res 2016;141:163-70.
